# PeAP1-mediated oxidative stress response plays an important role in the growth and pathogenicity of *Penicillium expansum*

**DOI:** 10.1128/spectrum.03808-22

**Published:** 2023-09-21

**Authors:** Yong Chen, Yichen Zhang, Dongying Xu, Zhanquan Zhang, Boqiang Li, Shiping Tian

**Affiliations:** 1 State Key Laboratory of Plant Diversity and Specialty Crops, Institute of Botany, Chinese Academy of Sciences, Beijing, China; 2 China National Botanical Garden, Beijing, China; 3 University of Chinese Academy of Sciences, Beijing, China; Yeungnam University, Gyeongsan, Gyeongbuk, South Korea

**Keywords:** *Penicillium expansum*, post-harvest blue mold, oxidative stress response, PeAP1 transcription factor, pathogenicity, transcriptome analysis

## Abstract

*Penicillium expansum* is the causal agent of post-harvest blue mold in various fruits and serves as a model for understanding fungal pathogenicity and mycotoxin production. The relevance of oxidative stress response in the growth and virulence of *P. expansum* has been largely unexplored. Here, we identify the transcriptional factor PeAP1 as a regulator of oxidative stress response in *P. expansum*. Gene expression and protein abundance of PeAP1, as well as its nuclear localization, are specifically induced by H_2_O_2_. Deletion of *PeAP1* results in increased sensitivity to H_2_O_2_, and *PeAP1* mutants exhibit a variety of defects in hyphal growth and virulence. PeAP1 prevents the accumulation of both intracellular H_2_O_2_ during vegetative growth and host-derived H_2_O_2_ during biotrophic growth. Application of an antioxidant glutathione and a NADPH oxidase inhibitor, diphenylene iodonium, to the *PeAP1* mutant partially restored fungal growth and virulence. RNA sequencing analysis revealed 144 H_2_O_2_-induced PeAP1 target genes, including four antioxidant-related genes, *PeGST1*, *PePrx1*, *PePrx2*, and *PeTRX2*, that were also demonstrated to be involved in oxidative stress response and/or virulence. Collectively, our results demonstrate the global regulatory role of PeAP1 in response to oxidative stress and provide insights into the critical role of the PeAP1-mediated oxidative stress response to regulate growth and virulence of *P. expansum*.

**IMPORTANCE:**

Reactive oxygen species are the core of host plant defense and also play a vital role in the successful invasion of host plants by pathogenic fungi. Despite its importance, the relevance of oxidative stress response in fungal growth and virulence is poorly understood in *P. expansum*. In this study, we reveal that the transcription factor PeAP1 acts as a central regulator of oxidative stress response in *P. expansum* and that there is a major link between PeAP1-mediated oxidative stress response and fungal growth and virulence. To explore the underlying mechanisms, we performed comparative transcriptomic studies and identified a number of H_2_O_2_-induced PeAP1 target genes, including four novel ones, *PePrx1*, *PePrx2*, *PeGST1*, and *PeTRX2*, whose functions were linked to PeAP1 and pathogenicity. These findings provide novel insights into the regulation mechanism of PeAP1 on growth and virulence, which might offer promising targets for control of blue mold and patulin contamination.

## INTRODUCTION

Plant pathogens must efficiently sense and adapt to many different host-induced stresses in order to survive (1). One of the most stressful factors is a host-induced oxidative burst, comprising the production of reactive oxygen species (ROS), including superoxide (O_2_^–^) and hydrogen peroxide (H_2_O_2_) generated by NADPH oxidases (2, 3). ROS accumulation at the site of pathogen invasion can either directly kill the pathogen or function as a secondary messenger, resulting in the induction of various plant defense-related genes (4 – 6). Pathogens have evolved multiple defense systems, both enzymatic and non-enzymatic, to cope with oxidative stress (7, 8). Several steps are involved in the activation of these defense systems, including the induced expression of transcription factors that regulate antioxidant response (9). For example, the *Saccharomyces cerevisiae* transcription factor YAP1 functions as a central regulator of oxidative stress response and controls the expression of various genes involved in ROS detoxification (10, 11). YAP1 contains three highly conserved domains: a basic leucine zipper (bZIP) domain important for DNA binding and two cysteine-rich domains at the N- and C-termini for redox regulation (12). YAP1 is localized in both the cytoplasm and the nucleus under normal conditions. In response to oxidative stress, however, the protein changes its conformation through the formation of two disulfide bonds and is relocated to the nucleus, where it activates the expression of oxidative stress-related genes (13, 14).

YAP1 homologs have been identified and characterized in several fungal pathogens, where they are usually involved in oxidative stress tolerance (15). For example, YAP1 in the maize pathogen *Ustilago maydis* mediates oxidative stress response and positively regulates fungal virulence (16). Studies have identified 203 YAP1-dependent genes that are upregulated in *U. maydis* in response to H_2_O_2_ stress, many of which have YAP1 binding sites (TTASTMA, TTACGTAA, and TKACAAA) in their promoters (13, 17, 18). A significant number of YAP1-activated genes are directly involved in the detoxification of ROS, including genes coding for haeme peroxidase, cytochrome C peroxidase, alkyl hydroperoxide reductase, glutathione (GSH)-S-transferase, and thioredoxin 2 (16). YAP1-mediated detoxification of ROS has been identified as an important virulence determinant in *Magnaporthe oryzae* (19), *Colletotrichum gloeosporioides* (20), and *Alternaria alternata* (21). Notably, disruption of the YAP1 homolog in the plant pathogens *Cochliobolus heterostrophus* (22) and *Botrytis cinerea* (23), as well as the animal pathogen *Aspergillus fumigatus* (24), did not alter fungal virulence. These findings indicate that the relationship between oxidative stress and virulence is varied among the fungal species. Details of the mechanisms by which YAP1 homologs regulate virulence in fungal pathogens, however, are not well understood.

*Penicillium expansum* is the causal agent of post-harvest blue mold in a number of fruits, resulting in substantial economic losses (25). Additionally, the occurrence of blue mold decay is frequently associated with contamination of the harvested or processed product with the mycotoxin patulin that causes serious health problems (26, 27). *P. expansum* is a necrotrophic fungal pathogen that infects wounds and secretes a range of virulence factors to kill host plant cells and acquire the nutrients released from the dead cells (28, 29). Virulence factors produced by *P. expansum* are strictly regulated during the infection process by a complex regulatory network involving multiple regulatory factors and control elements, which in turn respond to host signals (28). For example, the transcription factors PePacC, PeMetR, and PeCreA associated with broad responses to ambient pH, sulfur source, and carbon source, respectively, have been shown to affect the pathogenicity of *P. expansum* (30 – 32). ROS have also been shown to play an important role in fruit-microbe interactions (33). For example, in response to the initial colonization of *P. expansum* in a non-host citrus fruit, the host releases a significant amount of H_2_O_2_ at the infection site, inhibiting fungal colonization (34). The addition of the H_2_O_2_-scavenging enzyme catalase, however, can reduce H_2_O_2_ production in the non-host fruit and promote the successful colonization of *P. expansum*. These findings indicate a potential link between oxidative stress response and virulence in *P. expansum*. However, the mechanism by which *P. expansum* avoids the negative impact of host-induced ROS production after invasion remains poorly understood.

In the present study, we identify PeAP1 in *P. expansum* as a homolog of the bZIP transcription factor YAP1 and find that it mediates oxidative stress response and plays an important role in supporting hyphal growth and virulence. A number of H_2_O_2_-induced PeAP1 target genes were also identified through transcriptome analysis, and several of them were further demonstrated to be involved in oxidative stress tolerance and/or virulence.

## RESULTS

### PeAP1 is a YAP1 homolog in *P. expansum*

A BLASTP search of the *P. expansum* T01 genome database identified a bZIP transcription factor, which we named PeAP1, that displayed 59% and 22% overall identity with *Aspergillus nidulans* NapA (Q5AW17) and *S. cerevisiae* YAP1 (CAA41536), respectively. PeAP1 comprises 587 amino acids, an N-terminal bZIP DNA-binding domain (residues 147–208), and a C-terminal cysteine-rich domain (c-CRD, residues 516–579). Alignments of PeAP1 with other YAP1 homologs indicated that the bZIP domain and c-CRD domain in PeAP1 are highly similar to these domains in other fungi, having a conserved L-rich region (L174, L181, and L195) in the bZIP domain and a conserved C-rich region (C534, C558, and C567) in the c-CRD domain (Fig. S1A). A phylogenetic tree based on a full sequence comparison of YAP1-like proteins from different fungi revealed that the PeAP1 sequence is most distant from the yeast YAP1 and most similar to sequences from *Aspergillus* spp. (Fig. S1B). The domain characteristics and the phylogenetic tree analysis suggested that PeAP1 may have biological functions similar to YAP1-like proteins in other fungi.

### PeAP1 is essential for coping with oxidative stress

*PeAP1* disruption strains were constructed by homologous gene replacement to investigate the function of PeAP1 in *P. expansum* (Fig. S2A). Three separate targeted-deletion mutants, designated Δ*PeAP1*-1, Δ*PeAP1*-3, and Δ*PeAP1*-4, were created, verified by PCR (Fig. S2B), and further conﬁrmed by Southern blot analysis (Fig. S2C). Although only data for Δ*PeAP1*-3 and Δ*PeAP1*-4 are presented in most of the figures, the phenotypes of all three mutants were identical. A complementary strain, *PeAP1* ^C^, was also generated by re-introduction of *PeAP1* into the native gene locus to confirm the function of PeAP1.

**FIG 1 F1:**
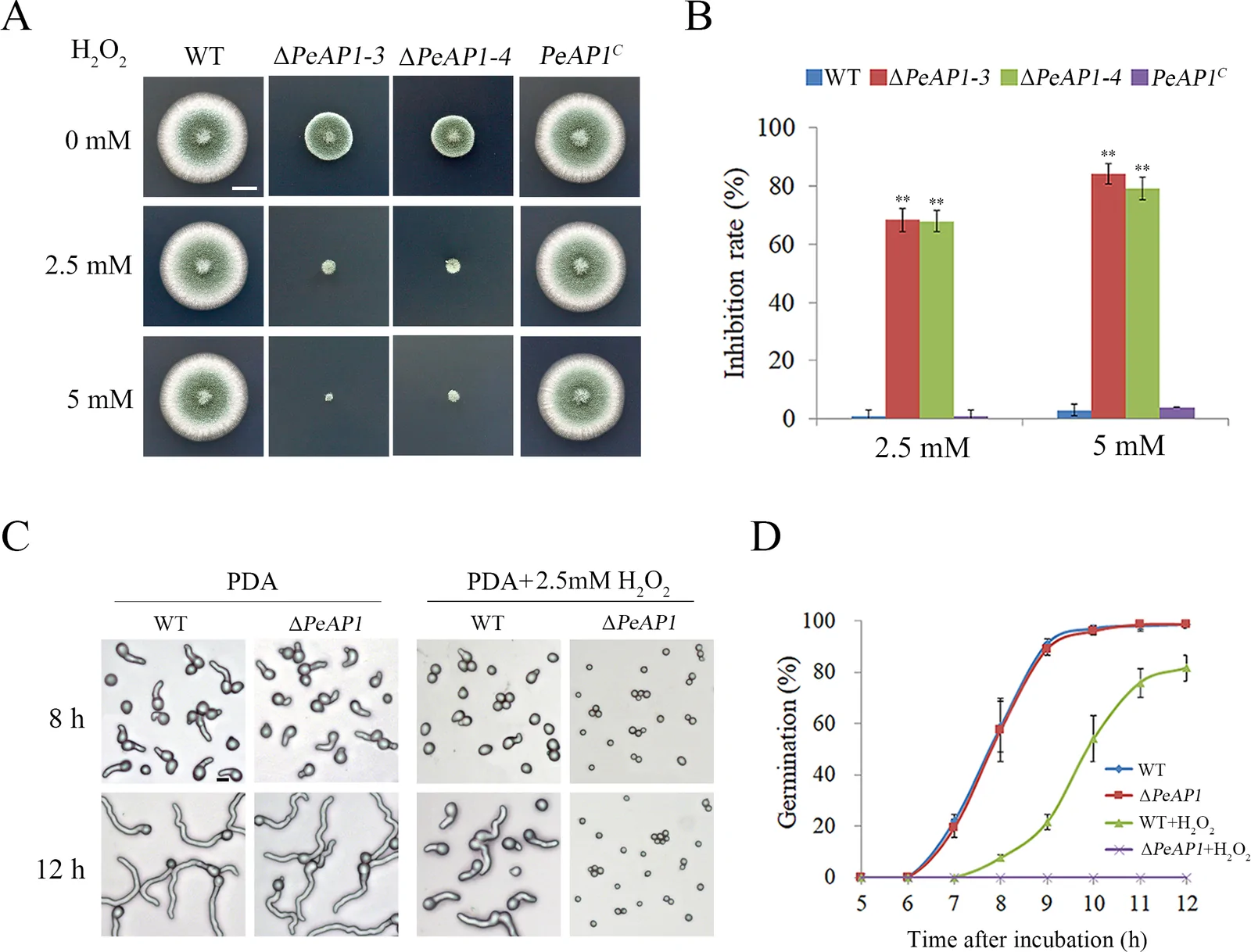
*PeAP1* deletion mutants of *P. expansum* exhibit hypersensitivity to H_2_O_2_. (**A**) Mycelial growth of the tested strains of *P. expansum* under oxidative stress conditions. WT, *PeAP1* mutant, and *PeMetR^C^* strains of *P. expansum* were cultured on PDA media with or without 2.5- or 5.0-mM H_2_O_2_ and cultured at 25°C for 4 days. Scale bar = 10 mm. (**B**) Rate of growth inhibition in the tested strains after 4 days of culture. Data represent the mean ± SEM (*n* = 3). **Significant difference at *P* < 0.01. (**C**) Conidial germination of tested strains of *P. expansum* under oxidative stress conditions. WT and Δ*PeAP1* strains were cultured on PDA media with or without 2.5 mM H_2_O_2_ at 25°C for 12 h. Scale bar = 10 µm. (**D**) Germination rates of the tested strains of *P. expansum* after 5–12 h of incubation. Data represent the mean ± SEM (*n* = 3). SEM, standard error of the mean.

Wild type (WT), *PeAP1* mutants, and *PeAP1* ^C^ strains were cultured on potato dextrose agar (PDA) media supplemented with the oxidant H_2_O_2_ to assess the role of PeAP1 in oxidative stress tolerance. Results indicated that the WT strain exhibited little difference in growth on H_2_O_2_-amended media, relative to growth on non-amended media. In contrast. mycelial growth in the *PeAP1* mutants was significantly inhibited at a level of about 70% and 80% when exposed to 2.5- and 5.0-mM H_2_O_2_, respectively (Fig. 1A and B). Germination of conidia also exhibited increased sensitivity to H_2_O_2_ in the *PeAP1* mutants. Conidia of *PeAP1* mutants exposed to 2.5-mM H_2_O_2_ did not swell or germinate up to 12 h after inoculation, while the germination rate of the WT strain was nearly 80% (Fig. 1C and D). The complementary strain, *PeAP1* ^C^, exhibited a level of tolerance to hydrogen peroxide similar to that of the WT strain (Fig. 1). These results indicate that PeAP1 is involved in oxidative stress response in *P. expansum* and contributes to ROS tolerance.

### Gene expression and protein abundance of PeAP1 are responsive to oxidative stress

**FIG 2:**
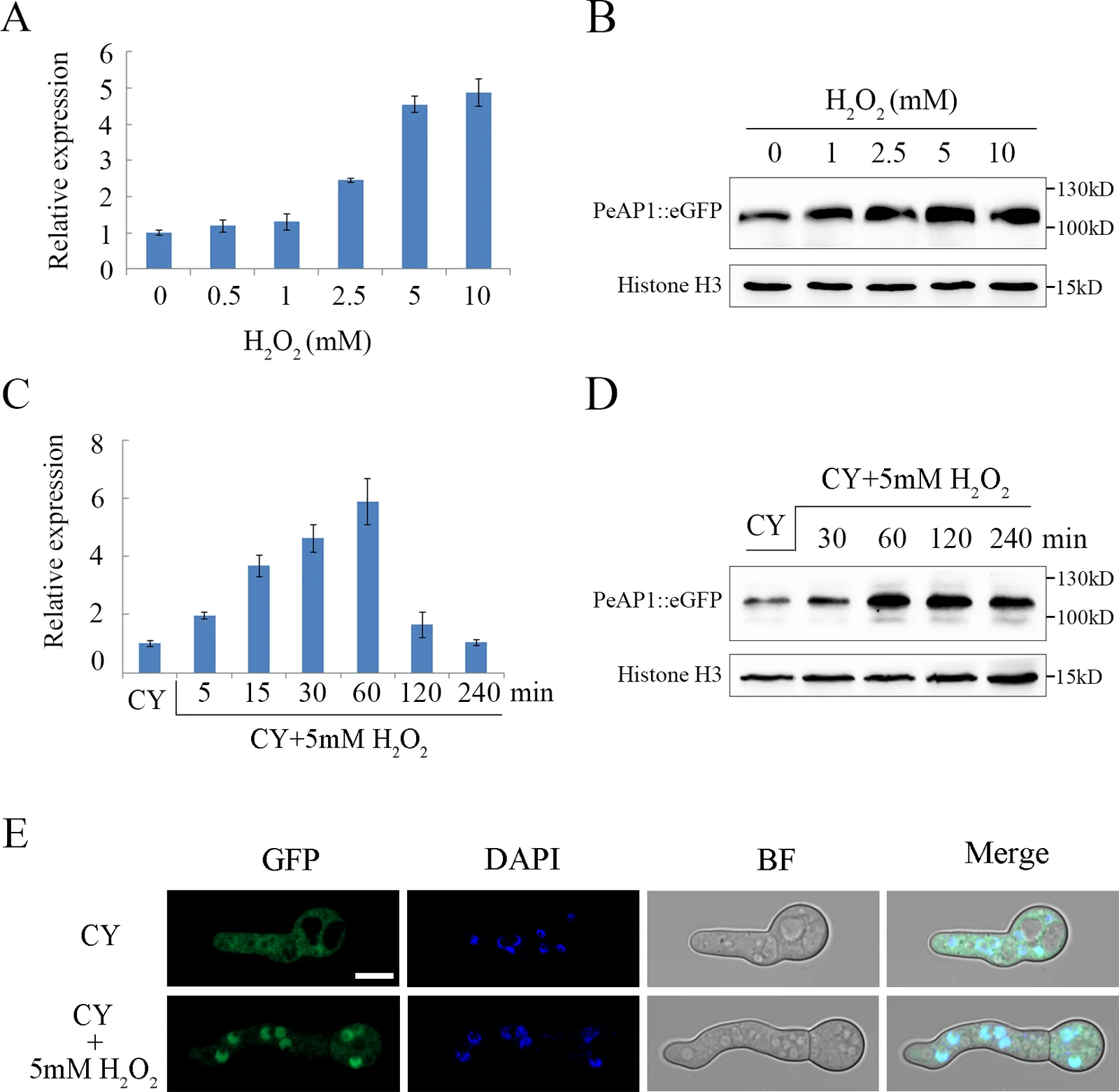
Gene expression, protein abundance, and subcellular localization of PeAP1 in *P. expansum* are H_2_O_2_ dependent. (**A**) Relative expression of PeAP1 in response to different concentrations of H_2_O_2_. Total RNA was isolated from WT strains cultured in CY media for 24 h and then transferred to CY media supplemented with 0.5, 1.0, 2.5, 5.0, or 10.0 mM of H_2_O_2_ for another 1 h. (**B**) Western blot analysis of PeAP1 abundance in *P. expansum* exposed to different concentrations of H_2_O_2_. Total cell extracts were isolated from Δ*PeAP1/PeAP1::eGFP* trains cultured in CY media for 24 h and then shifted to CY media containing 1.0, 2.5, 5.0, or 10.0 mM of H_2_O_2_ for another 2 h. (**C**) Relative expression of *PeAP1* in response to short-term exposure to H_2_O_2_ stress. Total RNA was isolated from the WT strain cultured in CY media for 24 h and then shifted to CY media containing 5 mM of H_2_O_2_ for 5, 15, 30, 60, 120, and 240 min. (**D**) Western blot analysis of PeAP1 in response to short-term exposure to oxidative stress. Total cell extracts were isolated from Δ*PeAP1/PeAP1::eGFP* trains cultured in CY media and then shifted to CY media containing 5 mM of H_2_O_2_ for 30, 60, 120, and 240 min. (**E**) Subcellular localization of PeAP1 in *P. expansum* treated with H_2_O_2_. The Δ*PeAP1/PeAP1::eGFP* strain was cultured in CY media for 14 h, exposed to 5-mM H_2_O_2_ for 2 h, and assessed microscopically for green fluorescence protein (GFP) fluorescence. Nuclei were visualized using the nuclear-specific stain, 4’,6-diamidino-2-phenylindole (DAPI). BF, bright field. Relative expression was normalized using histone H3 as an internal control. Data represent the mean ± standard error of the mean (*n* = 3). Histone H3 levels were used as a loading control in the Western blot analysis. Scale bar = 10 µm.

The level of *PeAP1* expression was analyzed in shift experiments to determine if it is involved in oxidative stress response in *P. expansum*. Mycelia of the WT strain were cultured in Czapek yeast (CY) media for 24 h, then shifted to media amended with different concentrations of H_2_O_2_ and incubated for an additional 1 h. Reverse transcription-quantitative PCR (RT-qPCR) analysis revealed that the relative level of *PeAP1* transcripts increased gradually with increases in the concentration of H_2_O_2_ (Fig. 2A), indicating that oxidative stress induced the expression of *PeAP1*.

A C-terminal *PeAP1::eGFP* fusion gene under the control of the endogenous *PeAP1* promoter was constructed and transformed into the Δ*PeAP1* mutant to further examine PeAP1 abundance under oxidative conditions. Results indicated that the Δ*PeAP1/PeAP1::eGFP* transformant exhibited no significant differences in growth in response to oxidative stress, relative to the WT strain (data not shown). This indicated that expression of the *PeAP1::eGFP* fusion gene rescued the defects present in the Δ*PeAP1* mutant. Mycelia of the Δ*PeAP1/PeAP1::eGFP* strain were grown overnight in CY media, then shifted to media supplemented with different concentrations of H_2_O_2_ and incubated for two additional hours. Western blot analysis with an anti-GFP antibody indicated that oxidative stress increased the abundance of PeAP1::eGFP fusion protein in a dose-dependent manner (Fig. 2B).

A shift experiment was also conducted to examine the short-term response of *PeAP1* to oxidative stress. Mycelia of WT strain were grown in CY media for 24 h and then shifted to media containing 5 mM of H_2_O_2_ for 5, 15, 30, 60, 120, and 240 min. RT-qPCR analysis indicated that *PeAP1* transcripts markedly increased when mycelia were exposed to H_2_O_2_, reaching a peak value at 60 min, then decreasing at 120 and 240 min (Fig. 2C). Similar trends in PaAP1 protein abundance were also observed (Fig. 2D). These results confirmed that both gene expression of *PeAP1* and protein abundance are H_2_O_2_ dependent.

### Nuclear localization of PeAP1 is modulated by H_2_O_2_

Culture of the Δ*PeAP1/PeAP1::eGFP* strain was shifted from CY media for 2 h to media supplemented with 5-mM H_2_O_2_ to determine if PeAP1 localization changes under oxidative stress conditions, as occurs in *S. cerevisiae* (35) and *U. maydis* (16). Results indicated that GFP fluorescence was distributed throughout the hyphal cytoplasm in the absence of oxidative conditions (Fig. 2E). Exposure to H_2_O_2_, however, resulted in the PeAP1::eGFP fusion protein being concentrated in nuclei, as determined by the colocalization of the eGFP and DAPI fluorescence signals. These results indicate that nuclear localization of PeAP1 is enhanced in response to oxidative stress and that the PeAP1 regulator functions as a redox sensor in *P. expansum*.

### PeAP1 is required for mycelial growth in *P. expansum*

**FIG 3 F3:**
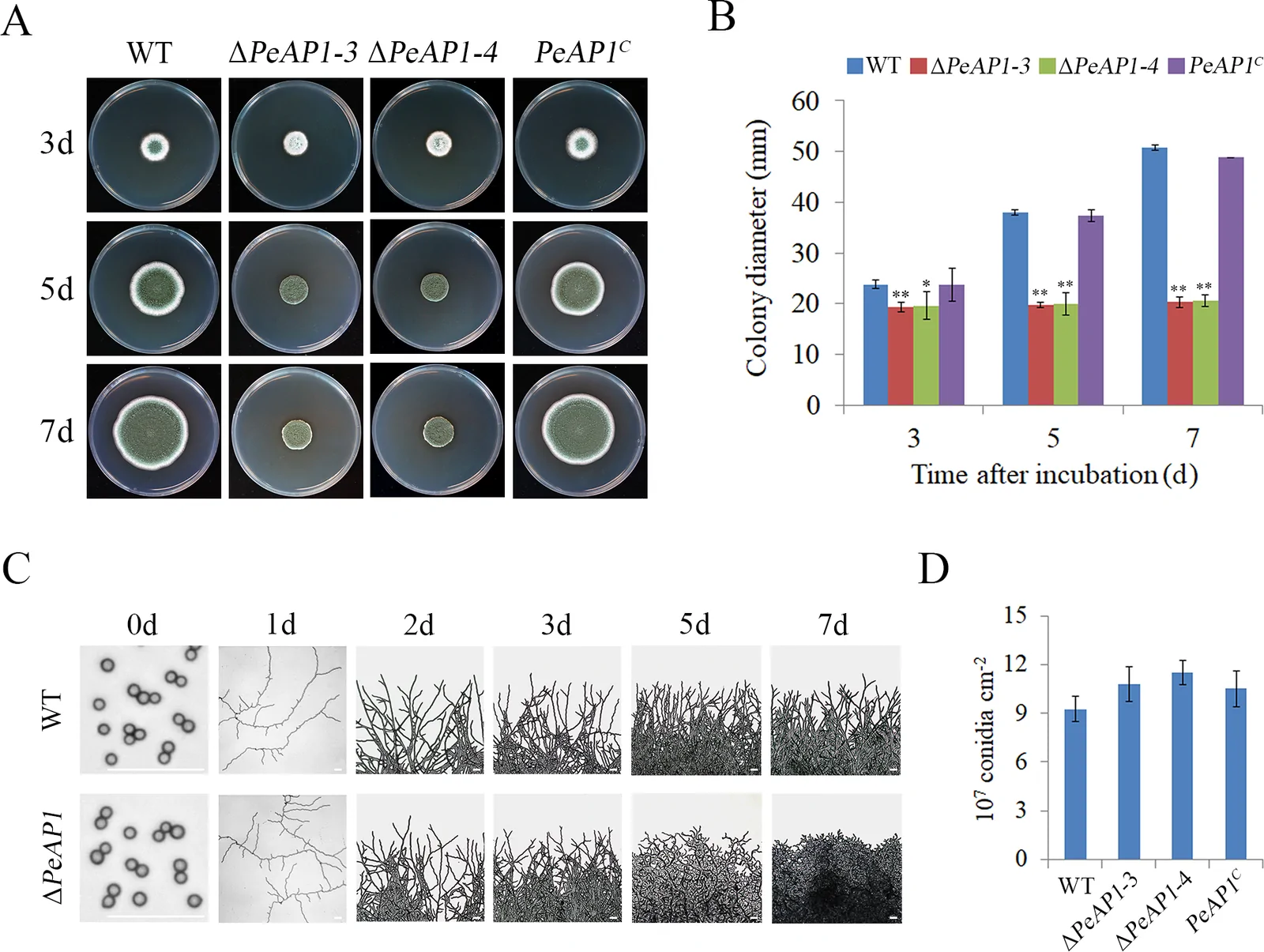
PeAP1 regulates vegetative growth in *P. expansum*. (**A and B**) Loss of *PeAP1* affects hyphal development and extension in PeAP1-deletion mutants of *P. expansum*. WT, *PeAP1* mutant, and *PeAP1^C^* strains of *P. expansum* were cultured on PDA media at 25°C for 10 days. Growth of the strains was photographically documented and quantitatively analyzed at 3, 5, and 7 dpi. (**C**) Deletion of *PeAP1* in *P. expansum* causes deformities in the hyphae of mutant strains observed at the edge of the growing colonies. Scale bar = 50 µm. (**D**) Loss of *PeAP1* of *P. expansum* does not affect conidiation in mutant strains. Data represent the mean ± standard error of the mean (*n* = 3). *Significant difference at *P* < 0.05, **significant difference at *P* < 0.01.

Conidial germination, hyphal development and extension, and conidiation in *PeAP1* mutants were observed over a time course of 10 days to evaluate the role of PeAP1 in the vegetative growth and vegetative reproduction of *P. expansum. PeAP1* mutants exhibited normal germination and growth on PDA media during the first 48 h of culture (Fig. 1C, D and 3C)[Fig F1 F3]. In contrast, colonies of the mutant strains stopped expanding after reaching a diameter of approximately 20 mm at 3 days post-inoculation (dpi), exhibiting about a 50% and 60% reduction in growth at 5 and 7 dpi, respectively, relative to the WT strain (Fig. 3A and B). A similar inhibition of growth of *PeAP1* mutants was also observed on CYA media (Fig. S3). Further microscopic examination of mycelia taken from the edge of Δ*PeAP1* colonies revealed hyphal deformities, tip swelling, and short branching after 3 days of culture (Fig. 3C). Notably, the level of conidiation in *PeAP1* mutants was comparable to the level of conidiation in the WT strain (Fig. 3D). Collectively, the results indicated that PeAP1 is required for vegetative growth but not for conidiation in *P. expansum*.

### PeAP1 prevents the accumulation of intracellular H_2_O_2_ in *P. expansum* during vegetative growth

**FIG 4:**
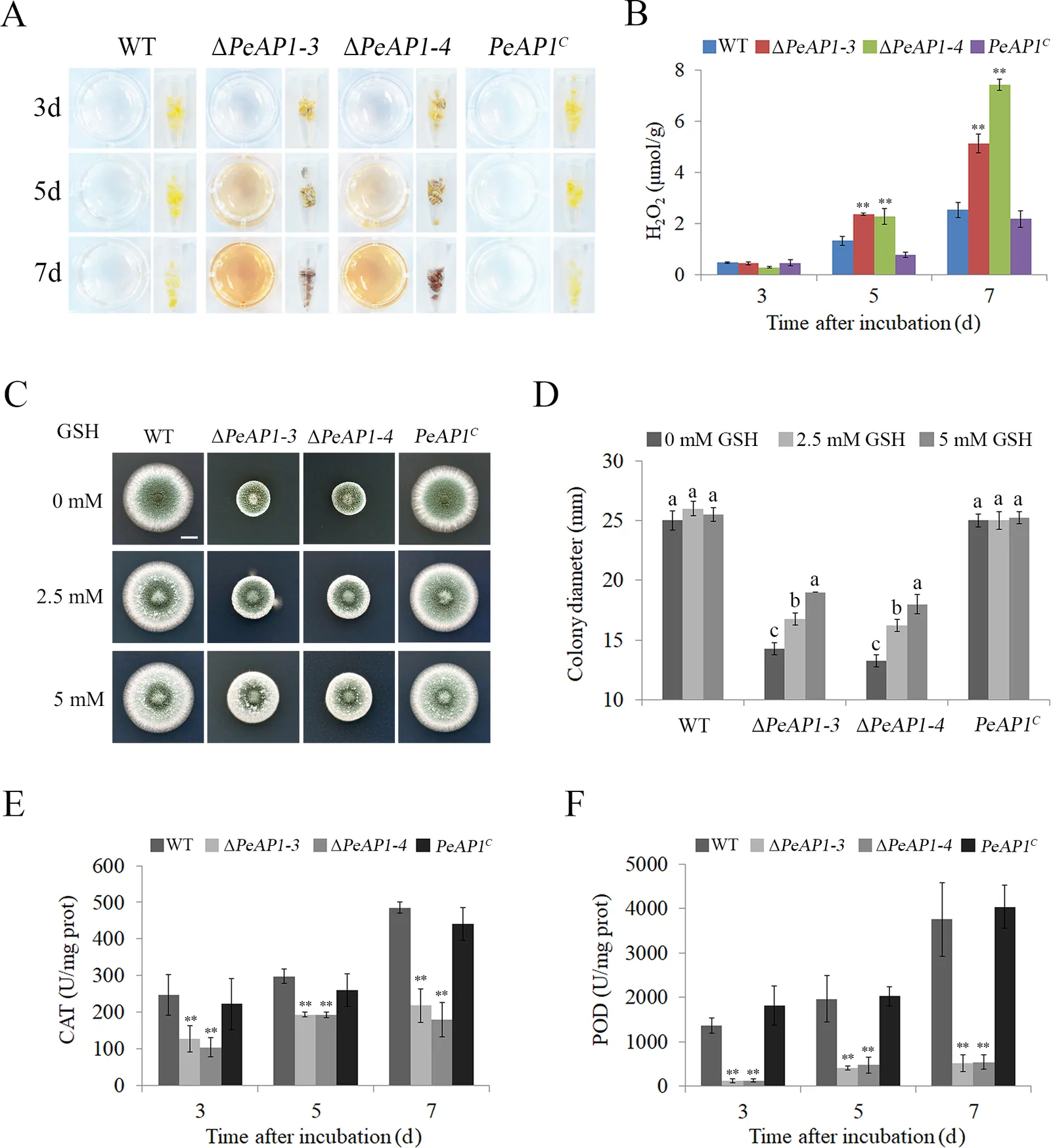
PeAP1 in *P. expansum* is required for maintaining H_2_O_2_ homoeostasis during vegetative growth. (**A and B**) The accumulation of intracellular H_2_O_2_ is affected in *PeAP1* mutants. WT, *PeAP1* mutant, and *PeAP1^C^* strains of *P. expansum* were cultured on PDA media at 25°C for 3, 5, and 7 days. The accumulation of intracellular H_2_O_2_ of the tested strains was qualitatively analyzed by DAB staining and quantitatively analyzed using a spectrophotometric method. Data represent the mean ± SEM (*n* = 3). **Significant difference at *P* < 0.01. (**C and D**) GSH partially restores the impaired growth of *PeAP1* mutants. WT, *PeAP1* mutant, and *PeAP1^C^* strains of *P. expansum* were cultured on PDA media supplemented with or without 2.5- or 5.0-mM GSH and grown at 25°C for 4 days. Growth of the strains was photographically documented and quantitatively analyzed. Data represent the mean ± SEM (*n* = 3). Columns with different letters are significantly different from each other as determined using a least significant difference test (*P* < 0.05). Scale bar = 5 mm. (**E and F**) PeAP1 in *P. expansum* regulates the activity of antioxidant enzymes. WT, *PeAP1* mutant, and *PeAP1^C^* strains of *P. expansum* were cultured on PDA media at 25°C for 3, 5, and 7 days. An identical quantity of fresh mycelia of each of the tested strains was used for the analysis of CAT and POD activity. Data represent the mean ± SEM (*n* = 3). **Significant difference at *P* < 0.01. SEM, standard error of the mean. CAT, catalase; POD, peroxidase.

Considering the increased sensitivity of *PeAP1* mutants to H_2_O_2_, two strategies were pursued to determine if H_2_O_2_ plays a role in the growth defects observed in deletion mutants. Firstly, diaminobenzidine (DAB) staining was used to examine the accumulation of intracellular H_2_O_2_ in the different *P. expansum* strains. Little staining was observed in colonies of both WT and *PeAP1* mutants at 33 dpi; however, colonies of the deletion mutants exhibited an intensive brown staining after 5 days of culture, indicating an increased accumulation of H_2_O_2_, relative to the WT or complemented strains (Fig. 4A). Quantitative analysis using a spectrophotometric method indicated a gradual accumulation of H_2_O_2_ in *P. expansum* strains as time of culture increased, and that *PeAP1* mutants had accumulated higher levels of H_2_O_2_ than WT strain after 5 days of culture (Fig. 4B), which was consistent with the results of the staining analysis. The second strategy involved the use of the antioxidant GSH, whose use was intended to protect the fungal strains against H_2_O_2_. When PDA media was supplemented with 2.5- and 5.0-mM GSH, the impaired growth of Δ*PeAP1* was partially restored (Fig. 4C and D), suggesting that H_2_O_2_-induced oxidative stress contributed to the growth defects observed in the *PeAP1* mutants.

The activity of two H_2_O_2_ detoxifying enzymes, catalase (CAT) and peroxidase (POD), produced in the two *PeAP1* mutants was measured and compared with the activity of these two enzymes in the WT strain to further discern the role of PeAP1 in intracellular H_2_O_2_ accumulation. Results indicated that the *PeAP1* mutants exhibited significantly lower enzyme activity than the WT strain (Fig. 4E and F), suggesting that PeAP1 plays an important role in regulating the degradation of H_2_O_2_ in *P. expansum*. Given the compensating effect of GSH in vegetative growth, we also determined the intracellular content of GSH and glutathione reductase (GR) activity. Results revealed lower GSH content and GR activity in the *PeAP1* mutants compared to the WT strain (Fig. S4), indicating that PeAP1 may also be involved in regulating the synthesis of the antioxidant GSH. Overall, the results suggest that impaired H_2_O_2_ detoxification in the Δ*PeAP1* mutant results in the accumulation of intracellular H_2_O_2_ in the mycelia of *P. expansum* with increasing time of culture, which in turn affects fungal hyphal development and extension.

### PeAP1 is a virulence determinant in *P. expansum*

**FIG 5:**
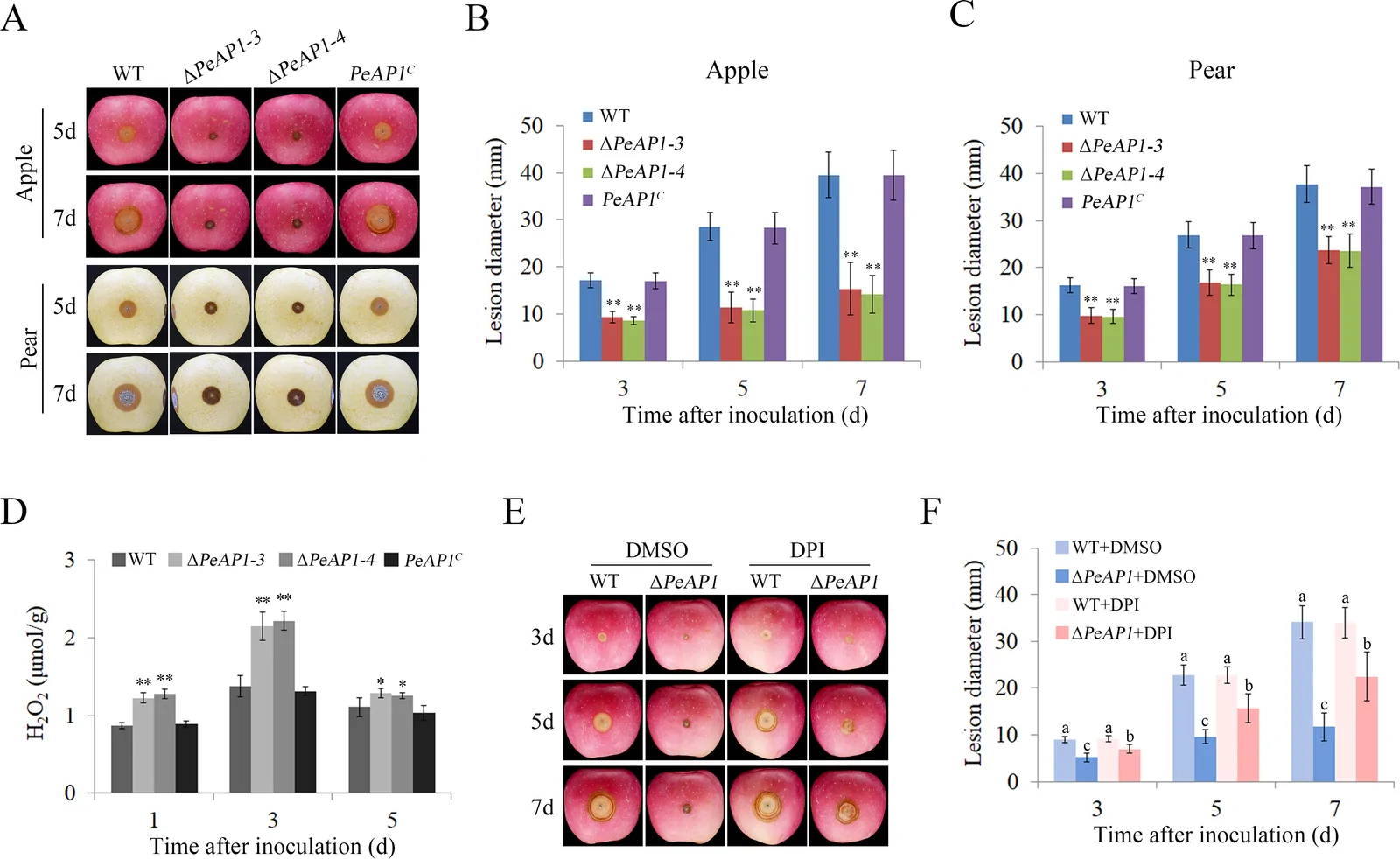
PeAP1 plays an essential role in the pathogenicity *of P. expansum*. (**A through C**) *PeAP1* mutants exhibit significantly reduced levels of virulence in fruit hosts. Time course of decay development in apple and pear fruits inoculated with conidia of WT, *PeAP1* mutant, and *PeAP1^C^* strains of *P. expansum*. Photographs were taken at 5 and 7 dpi. Diameters of lesions on apple fruit and pear fruit were measured at 3, 5, and 7 dpi. Data represent the mean ± SEM (*n* = 3). **Significant difference at *P* < 0.01. (**D**) *PeAP1* mutants exhibit increased H_2_O_2_ accumulation in host tissues at the site of inoculation during biotrophic growth. Conidia of WT, *PeAP1* mutant, and *PeAP1^C^* strains of *P. expansum* were pipetted into wounds made in apple fruit. Fruits were stored at 25°C for 5 days. Identical quantities of host tissues taken from the disease-health junction at 1, 3, and 5 dpi were used for the analysis of H_2_O_2_ accumulation using a spectrophotometric method. Data represent the mean ± SEM (*n* = 3). *Significant difference at *P* < 0.05; ** significant difference at *P* < 0.01. (**E and F**) DPI partially restores the virulence of *PeAP1* mutant strains of *P. expansum*. Time course of decay development in apple fruit inoculated with conidia of WT and Δ*PeAP1* strains along with dimethyl sulfoxide (DMSO) or DPI dissolved in DMSO. The infection process in the strains was photographically documented and quantitatively analyzed at 3, 5, and 7 dpi. Data represent the mean ± SEM (*n* = 3). Columns with different letters are significantly different from each other as determined by a least significant difference test (*P* < 0.05).

Conidial suspensions of WT, Δ*PeAP1* mutants, and *PeAP1^C^* strains were inoculated on apple fruit to investigate the role of PeAP1 in the pathogenicity of *P. expansum*. Blue mold lesions caused by *PeAP1* mutants were significantly smaller than those caused by the WT strain. Only small dark brown necrotic spots were observed at the sites of Δ*PeAP1* inoculation at 5 dpi, with only minor increases in lesion size observed on subsequent days (Fig. 5A and B). A decrease in virulence was also observed on pear fruit,with only small lesions being observed with the *PeAP1* mutants (Fig. 5A and C). Notably, the reduction in virulence was apparently eliminated by re-introducing the *PeAP1* gene into a *PeAP1* mutant (Fig. 5A through C). These results indicate that PeAP1 is an important determinant of pathogenicity in *P. expansum*.

### Increased H_2_O_2_ accumulation contributes to the reduced virulence of *PeAP1* mutants

H_2_O_2_ production around inoculation sites in apple fruit was assessed using a spectrophotometric method to gain insight into the mechanism responsible for the impaired pathogenicity of *PeAP1* mutants. High levels of H_2_O_2_ were detected in samples inoculated with *PeAP1* mutants during the first 3 dpi compared to apple wounds inoculated with WT and *PeAP1* ^C^ strains (Fig. 5D). H_2_O_2_ concentration in all fruit samples decreased at 5 dpi; however, H_2_O_2_ levels in sites inoculated with *PeAP1* mutants were still higher than they were in WT-infected sites. These results suggest that PeAP1 functions in preventing the accumulation of H_2_O_2_ around the inoculation site of fruits during the early stages of biotrophic growth.

DPI, an NADPH oxidase inhibitor, was administered to apple wounds together with spores of the different *P. expansum* strains to determine if the *PeAP1* mutants were able to infect the fruit more efficiently if H_2_O_2_ production and accumulation were impaired. Notably, the virulence of *PeAP1* mutants was partially restored when apple fruit wounds were treated with DPI (Fig. 5E and F). Similar results were also obtained in wounded/inoculated grape berries (Fig. S5). These results support the premise that H_2_O_2_ accumulation in wound sites contributes to the impaired pathogenicity of the *PeAP1* mutants.

### PeAP1 modulates transcriptional response to oxidative stress

**FIG 6:**
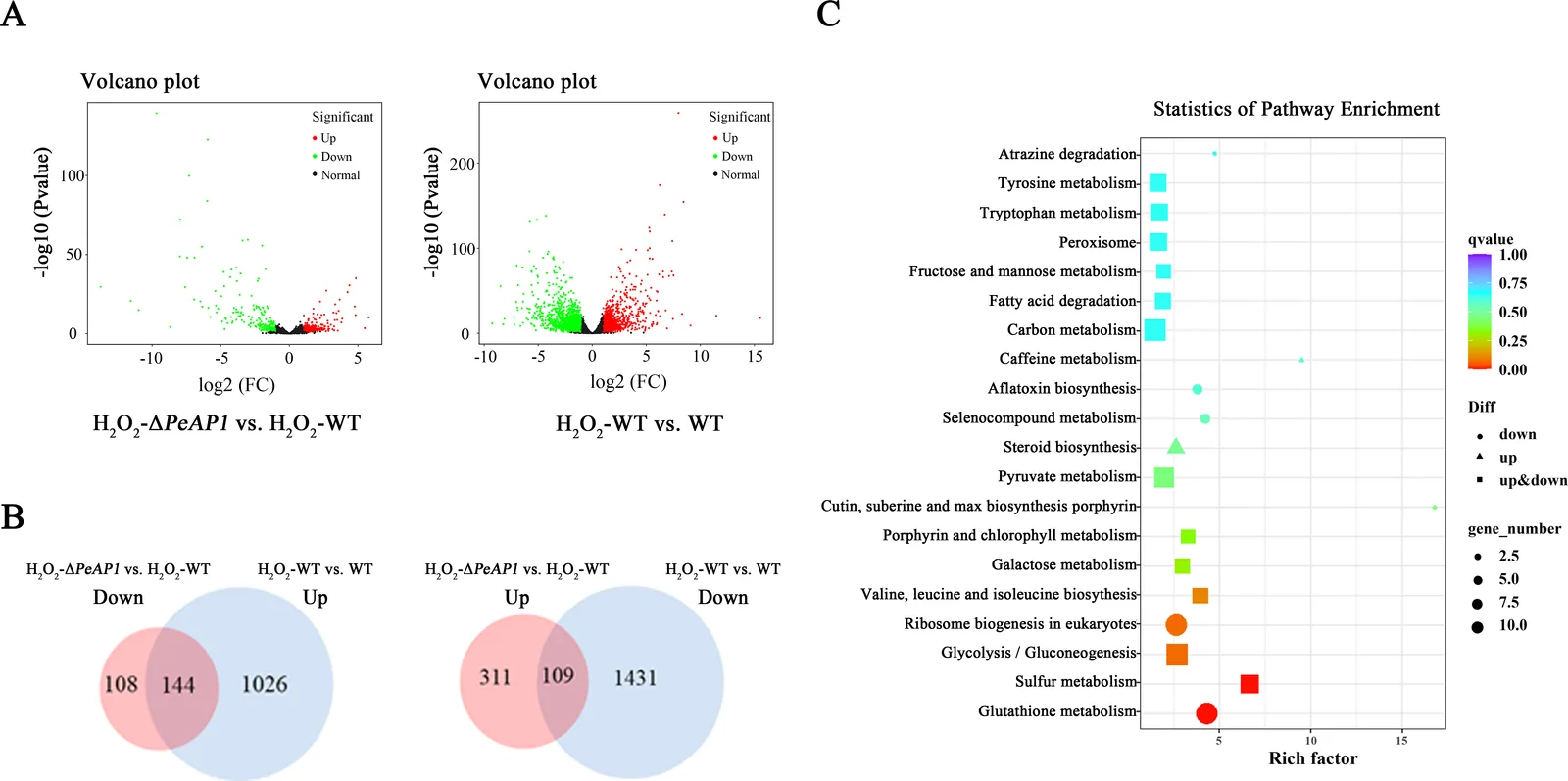
Identification of H_2_O_2_-induced PeAP1 target genes in *P. expansum* by RNA sequencing. (**A**) Volcano plot of gene expression patterns in a Δ*PeAP1* mutant and WT strains of *P. expansum* in response to an H_2_O_2_ treatment. (**B**) Venn diagram of DEGs in Δ*PeAP1* mutant and WT strains of *P. expansum* in response to an H_2_O_2_ treatment. (**C**) Scatter plot of KEGG pathway enrichment statistics of DEGs in Δ*PeAP1* mutant strains of *P. expansum* in response to oxidative stress.

Transcript profiles of WT and Δ*PeAP1* strains were generated by RNA sequencing (RNA-seq) from mycelia grown in CY media in the absence or presence of H_2_O_2_ to provide a broader insight into the functions of PeAP1, especially those related to oxidative stress response. Statistical tests were performed to identify differentially expressed genes (DEGs) in two comparisons: H_2_O_2_-Δ*PeAP1* vs H_2_O_2_-WT to identify DEGs associated with PeAP1 under oxidative stress conditions, and H_2_O_2_-WT vs WT to identify DEGs associated with the response of wild-type *P. expansum* to H_2_O_2_. Volcano plots of gene expression patterns revealed that H_2_O_2_ had a more pronounced effect on genome-wide gene expression than the loss of *PeAP1* (Fig. 6A). In total, 2,710 genes displayed an H_2_O_2_-responsive pattern in the WT background: 1170 genes showed H_2_O_2_-induced expression and 1,540 genes showed H_2_O_2_-inhibited expression (Fig. 6B). In addition, 672 DEGs were identified in the Δ*PeAP1* vs WT comparison under H_2_O_2_ conditions with 420 genes being upregulated and 252 genes being downregulated (Fig. 6B). The Kyoto Encyclopedia of Genes and Genomes (KEGG) pathway enrichment analysis revealed that the DEGs in the H_2_O_2_-Δ*PeAP1* vs H_2_O_2_-WT comparison were associated with diverse pathways, including glutathione metabolism, peroxisome, sulfur metabolism, glycolysis, and ribosome biogenesis, which may play important functions in fungal oxidative stress response, growth, and pathogenicity (Fig. 6C).

**FIG 7:**
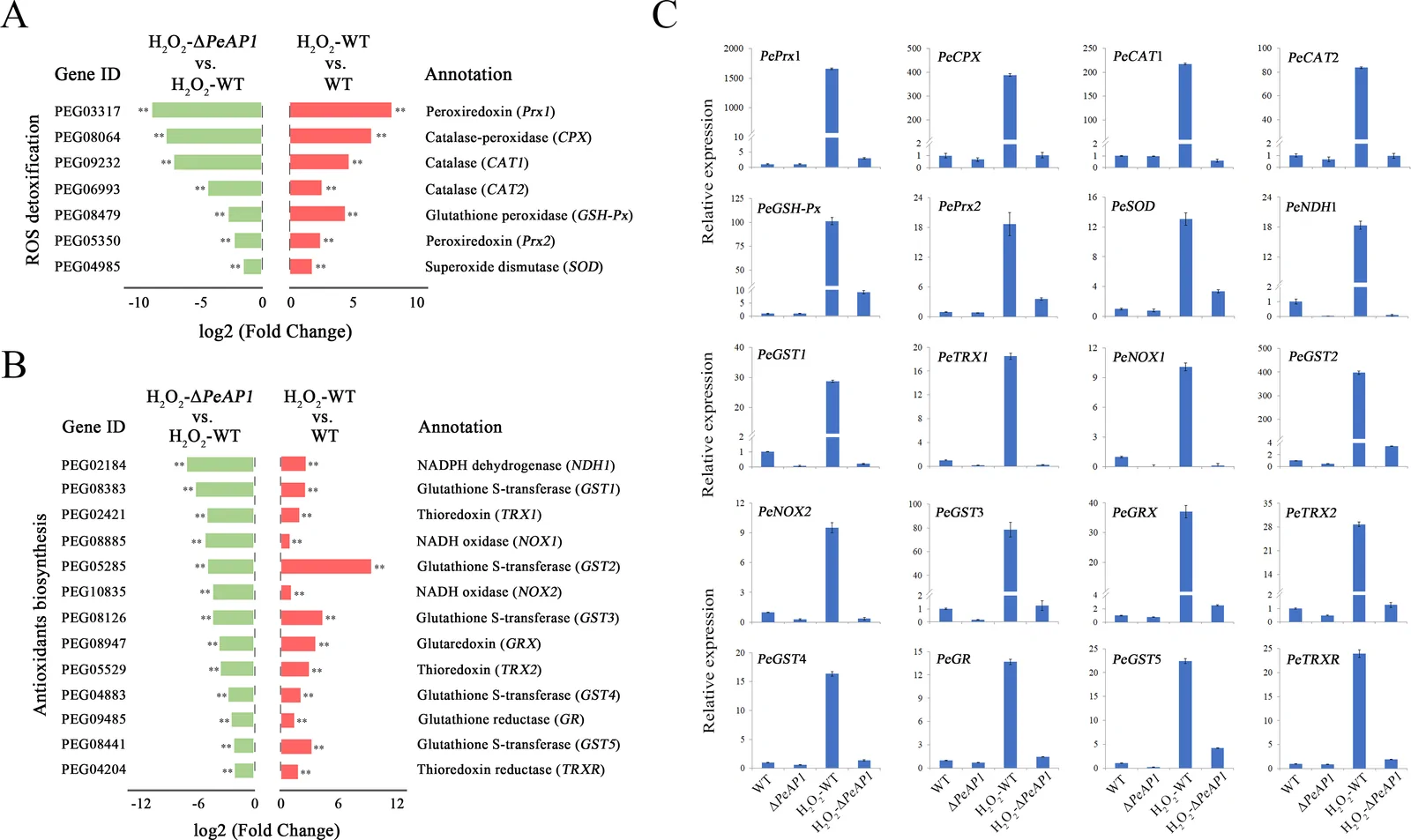
PeAP1 regulates the expression of multiple antioxidant-related genes. (**A and B**) Expression pattern of DEGs involved in ROS detoxification and antioxidant biosynthesis in Δ*PeAP1* mutant and WT strains of *P. expansum* in response to an H_2_O_2_ treatment. **Significant difference at *P* < 0.01. (C) Verification of the DEGs involved in antioxidant metabolism by RT-qPCR. Primers used are listed in Table S3. Relative expression was normalized using β-tubulin as an internal control. Data represent the mean ± SEM (*n* = 3).

RNA-seq data also revealed that the expression level of 144 H_2_O_2_-induced genes was downregulated in *PeAP1* mutants when exposed to H_2_O_2_ (designated as H_2_O_2_-induced PeAP1 target genes) (Fig. 6B; Table S2). Putative PeAP1 binding sites were found in the 1.5-kb promoter regions of 98 of these genes (Table S2). Regarding oxidative stress, 20 H_2_O_2_-induced PeAP1 target genes whose products participate in ROS detoxification (7 genes) and antioxidant biosynthesis (13 genes) were of special interest. Genes putatively involved in ROS detoxification include two peroxiredoxins (*Prx1* and *Prx2*), two catalases (*CAT1* and *CAT2*), a catalase-peroxidase (*CPX*), a glutathione peroxidase (*GSH-Px*), and a superoxide dismutase (*SOD*) (Fig. 7A). PeAP1 target genes involved in antioxidant biosynthesis include five glutathione S-transferases (*GST1-5*), a *GR*, a glutaredoxin (*GRX*), a thioredoxin reductase (*TRXR*), two thioredoxins (*TRX1* and *TRX2*), two NADPH dehydrogenase (*NDH1* and *NDH2*), and two NADH oxidases (*NOX1* and *NOX1*) (Fig. 7B). The expression pattern of these antioxidant-related genes was further assessed by RT-qPCR. Results indicated that the pattern of expression of all 20 genes was consistent with transcript levels in the RNA-seq data, despite any discrepancy in fold change (Fig. 7C).

### PeAP1 regulates the expression of four antioxidant-related genes involved in pathogenicity

**FIG 8:**
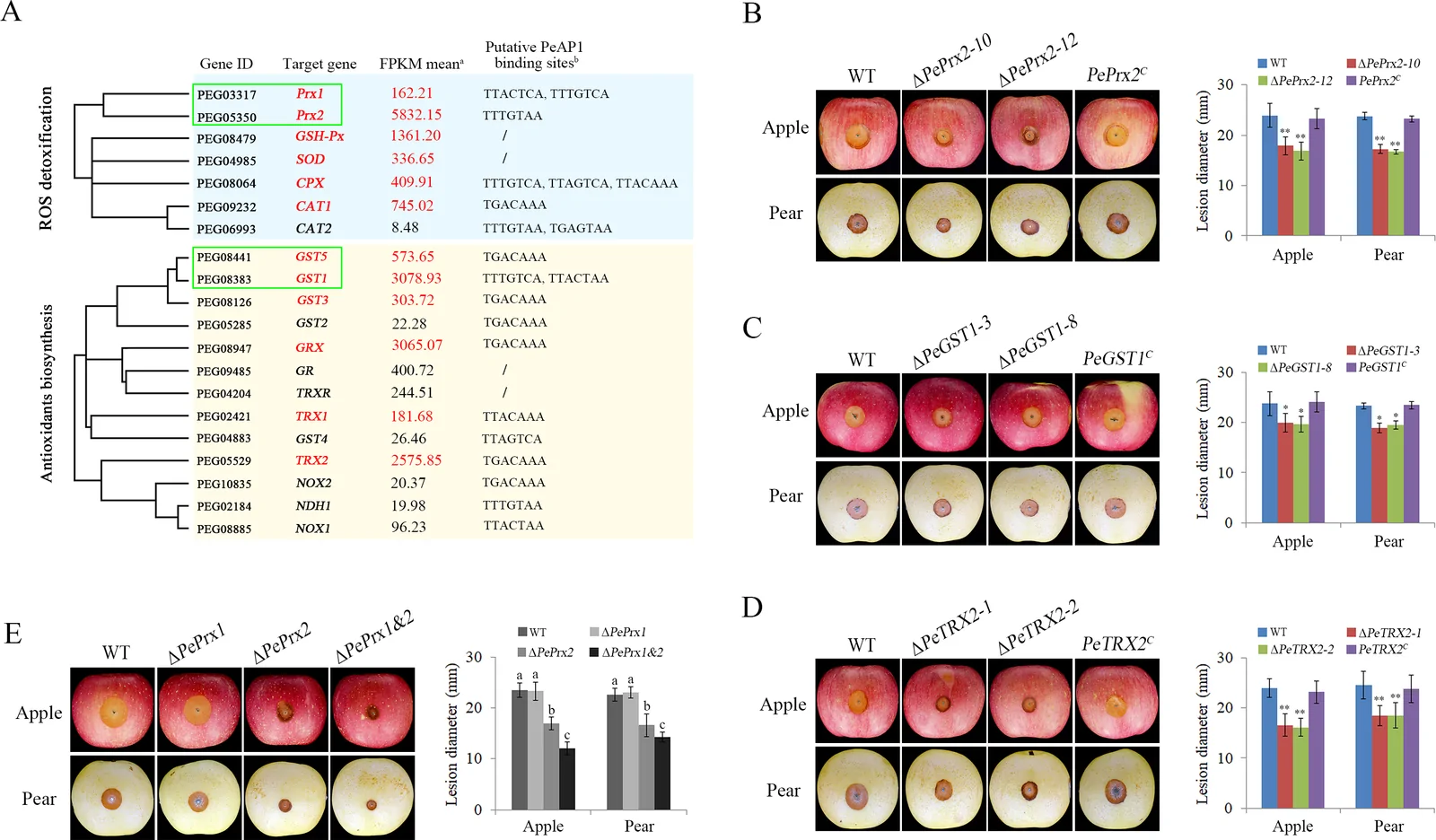
Four PeAP1-regulated genes are involved in virulence of *P. expansum*. (**A**) Twelve highly expressed genes in the antioxidant pathway whose expression was significantly downregulated in Δ*PeAP1* strains of *P. expansum* were selected for gene deletion. Phylogenetic trees based on a comparison of the full sequences of antioxidant-related enzymes were created with MEGA version 5.0 software using an ML method. ^a^FPKM values represent the average gene expression level in the WT strain exposed to H_2_O_2_. ^b^Sequences in the column represent the putative PeAP1 binding site identified in the promoter region of the respective gene. (**B through D**) Single‐gene deletion mutants in *P. expansum* of *PePre2*, *PeGST1*, and *PeTRX2* exhibit significantly reduced virulence in fruit hosts. Conidia of the tested strains were pipetted into wounds made in apple and pear fruit after, which fruit was incubated at 25°C for 7 days. Photographs were taken at 5 dpi. Diameters of the lesions were measured at 5 dpi. Data represent the mean ± SEM (*n* = 3). *Significant difference at *P* < 0.05; **significant difference at *P* < 0.01. (**E**) Δ*PePrx1&2* double mutants exhibited a phenotype with greater reductions in virulence than any single knockout mutant of *PePrxs*. Columns with different letters are significantly different from each other, using the least significant difference test (*P* < 0.05). ML, maximum likelihood.

To further confirm that PeAP1-mediated oxidative stress response accounts for the reduced virulence of *PeAP1* mutants, we characterized the function of 12 highly expressed genes (FPKM >100) in the antioxidant pathway whose expression was severely downregulated in the Δ*PeAP1* mutants when exposed to H_2_O_2_, among which 10 contain putative PeAP1 binding sites (Fig. 8A). These genes included *PePrx1*, *PePrx2*, *PeGSH-Px*, *PeSOD*, *PeCPX*, and *PeCAT1* in the ROS detoxification pathway and *PeGST1*, *PeGST5*, *PeGST3*, *PeGRX*, *PeTRX1*, and *PeTRX2* in the antioxidant biosynthesis pathway (Fig. 8A). Individual deletion mutants of these 12 genes were constructed and screened by PCR assay (Fig. S6). An evaluation of hyphal growth and conidiation indicated that the Δ*PePrx2* strain exhibited abnormal mycelial morphology and reduced mycelial growth when cultured on PDA but that conidiation was not affected. In contrast, the deletion of *PeTRX2* stimulated mycelial growth but significantly inhibited conidiation (Fig. S7A through C). Notably, both mutant strains exhibited reduced virulence on pear and apple fruit (Fig. 8B and D). Disease development using these two deletion mutants was reduced by about 30% at 5 dpi, compared to disease development with the WT strain. Disruption of *PeGST1* also resulted in attenuated virulence on fruit but did not affect mycelial growth and conidiation *in vitro* (Fig. 8C; Fig. S7A through C). Deletion of the other nine genes did not alter mycelial morphology or pathogenicity (Fig. S7).

Several enzymes in the antioxidant pathway are encoded by multiple genes (36). In this regard, two *Prx*’s, two *CAT*s, five *GST*s, two *TRX*s, two *NDH*s, and two *NOX*s were identified as H_2_O_2_-induced PeAP1 target genes in our RNA-seq data (Fig. 8A). Considering potential functional redundancy, we generated double mutants of *PePrx1*, *PePrx2* (Δ*PePrx1&2*) and *PeGST1*, *PeGST5* (Δ*PeGST1&5*) (Fig. S6). As predicted from our phylogenetic data, the Δ*PePrx1&*2 double mutants exhibited a phenotype with more conspicuous reductions in mycelial growth and virulence than any single knockout mutant of *PePrxs* (Fig. 8E; Fig. S7A through C), suggesting that *PePrx1* also plays an important role in fungal growth and pathogenicity. A different result was observed for the Δ*PeGST1&*5 strains, however, where no apparent defect in mycelial growth was observed, but virulence was reduced as was previously observed for the Δ*PeGST1* strain (data not shown). These results suggest that *PeGST5* is not directly involved in the virulence of *P. expansum*.

### *PePrx1*, *PePrx2*, and *PeTRX2* are involved in oxidative stress response

**FIG 9:**
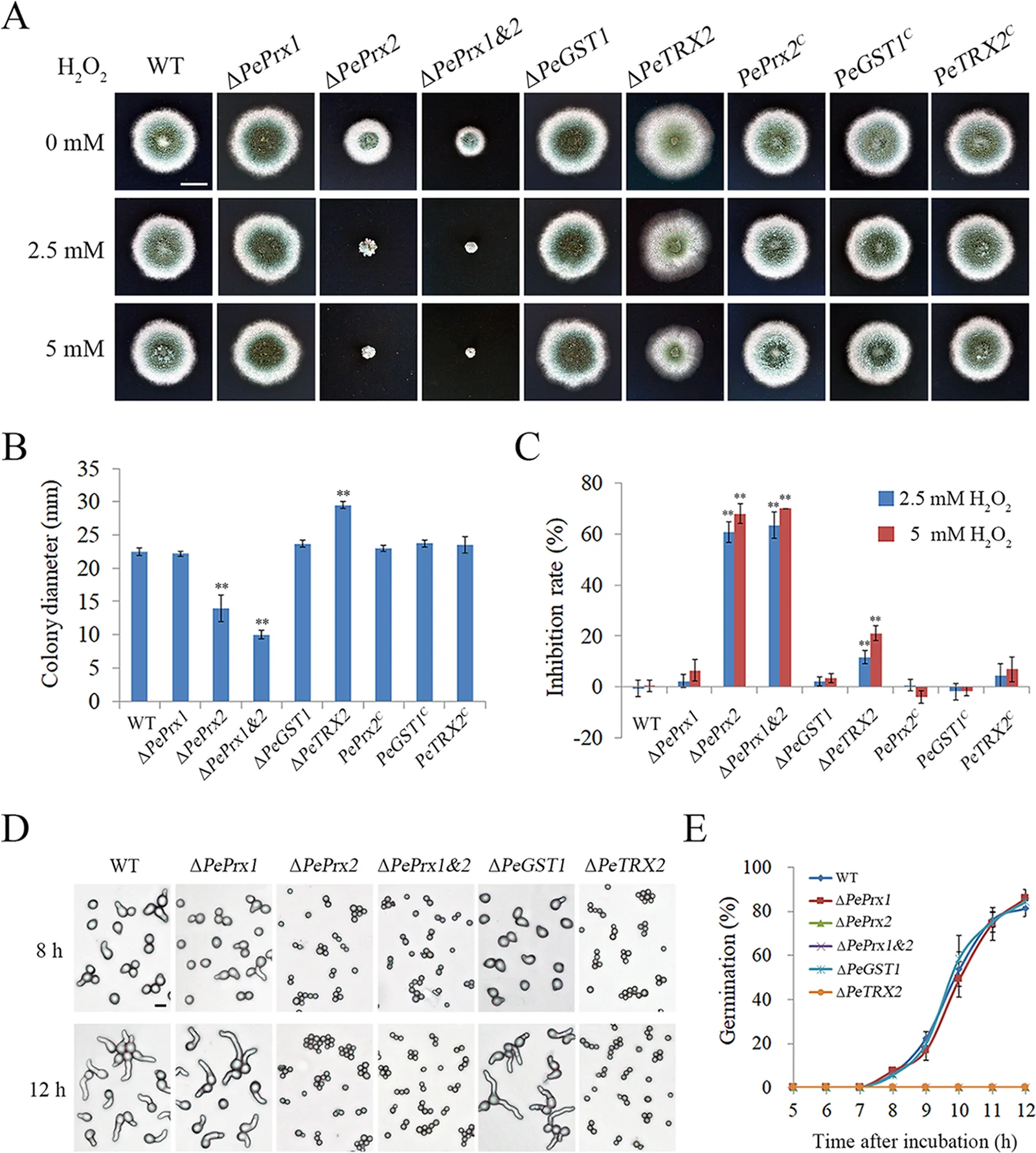
*PePrx1*, *PePrx2*, and *PeTRX2* are hypersensitive to H_2_O_2_. (**A**) Mycelial growth in tested strains grown under oxidative stress condition. The tested strains were inoculated on PDA media amended with or without 2.5- or 5.0-mM H_2_O_2_ at 25°C for 4 days. Scale bar = 10 mm. (**B and C**) Colony diameter and growth inhibition rate in tested strains at 4 dpi. Data represent the mean ± SEM (*n* = 3). **Significant difference at *P* < 0.01. (**D**) Conidial germination of the tested strains under oxidative stress conditions. The tested strains were inoculated on PDA media amended with or without 2.5-mM H_2_O_2_ and incubated at 25°C for 12 h. Scale bar = 10 µm. (E) Germination rate of the tested strains after 5–12 h of incubation. Data represent the mean ± SEM (*n* = 3).

Deletion mutant strains of *PePrx1*, *PePrx2*, *PeGST1*, and *PeTRX2* were incubated on PDA media supplemented with 2.5- or 5.0-mM H_2_O_2_ to assess if these putative PeAP1 target genes play an active role in oxidative stress tolerance. Results indicated that Δ*PePrx2* and Δ*PeTRX2* mutants, as well as Δ*PePrx1&*2 double mutants, were more sensitive than the WT strain to H_2_O_2_ (Fig. 9A through C). Mycelial growth in Δ*PePrx2* and Δ*PeTRX2* mutants was severely inhibited on PDA media amended with 5-mM H_2_O_2_, exhibiting a 67.9% and 21.1% rate of growth reduction, respectively, both of which were greater than the inhibition exhibited in the WT strain (Fig. 9C). Furthermore, the Δ*PePrx1&*2 double mutants exhibited higher sensitivity to H_2_O_2_ than either the single knockout mutant of *PePrxs*, having an average 63% reduction in mycelial growth when exposed to 2.5-mM H_2_O_2_ and 70% when exposed to 5.0-mM H_2_O_2_ (Fig. 9A through C). In contrast, the Δ*PePrx1* and Δ*PeGST1* mutants were not sensitive to H_2_O_2_.

The involvement of putative PeAP1 target genes in H_2_O_2_ tolerance was further confirmed by assessing spore germination in the deletion mutants. Notably, spores of the Δ*PePrx2*, Δ*PeTRX2*, and Δ*PePrx1&*2 strains were unable to swell or germinate in the presence of 2.5-mM H_2_O_2_ up to 12 h after exposure. In contrast, germination rates in Δ*PePrx1* and Δ*PeGST1* strains were not significantly different from the WT, reaching nearly 80% after 12 h of incubation (Fig. 9D and E). Collectively, these data suggest that similar to PeAP1, *PePrx1*, *PePrx2*, and *PeTRX2* also play an important role in oxidative stress tolerance in *P. expansum*.

## DISCUSSION

*P. expansum* is the causal agent of blue mold and produces the mycotoxin patulin (26, 37). Establishing effective disease management approaches to control *P. expansum* can often be problematic, despite its economic impact on fruit production and processed foods (25). Therefore, understanding the molecular basis for pathogenicity in *P. expansum* would greatly facilitate the design and development of an effective management strategy for blue mold decay (28). In the present study, we investigate PeAP1 in *P. expansum* as a homolog of the YAP1 transcription factor and find that it is involved in regulating both fungal growth and pathogenicity.

### PeAP1 acts as an important regulator of the oxidative stress response in *P. expansum*

Nuclear localization of the YAP1 transcription factor is a crucial step for YAP1 function in *S. cerevisiae* subjected to oxidative stress (35). Additionally, intramolecular disulfide bond formation by two cysteine residues from the c-CRD and n-CRD regions is thought to be necessary for nuclear translocation to occur (14). Similar to YAP1 in *S. cerevisiae*, PeAP1 in *P. expansum* possesses the conserved domains and cysteine residues (Fig. S1A). This suggests that PeAP1 may also have the capacity to create disulfide bonds and undergo nuclear localization. Indeed, nuclear localization of PeAP1 was observed in *P. expansum* under H_2_O_2_ stress conditions (Fig. 2E). The H_2_O_2_-induced nuclear localization of PeAP1 corresponds to the fact that the *PeAP1* deletion mutants were sensitive to H_2_O_2_ (Fig. 1) and the gene expression and protein abundance of PeAP1 were significantly increased in the WT strain after H_2_O_2_ treatment (Fig. 2A through D). These data suggest that PeAP1 is essential for increased tolerance to oxidative stress in *P. expansum*.

A comparative transcriptome analysis was conducted to examine in more detail the role of PeAP1 in the oxidative stress response. The majority of downregulated genes (57%) in the Δ*PeAP1* mutant exhibited H_2_O_2_-induced expression in the WT strain (Fig. 6B). Notably, disruption of PeAP1 resulted in decreased expression of many antioxidant-related genes, including 7 genes involved in ROS detoxification and 13 genes involved in antioxidant biosynthesis (Fig. 7). These findings indicate that PeAP1 is an important modulator of the transcriptional response to oxidative stress.

### PeAP1 prevents the accumulation of intracellular H_2_O_2_ during vegetative growth

The regulation of ROS homeostasis in filamentous fungi is an important factor in many aspects of fungal life including fungal growth and development. In this regard, YAP1-mediated ROS detoxification appears to play a major role in maintaining ROS homeostasis (38). Fungal pathogens generate ROS during conidial germination, hyphal tip growth, and the development of infection structures (39 – 41). Disruption of *MoAP1* in *M. oryzae* resulted in abnormal conidial morphology and a reduction in conidia formation (19). Similarly, deletion of *NapA* in *A. nidulans* resulted in reduced conidiation and the downregulation of 201 genes in conidia (42). However, *PeAP1* knockout mutants in our study displayed similar levels of conidiation to the WT strain (Fig. 3D), which is similar to the situation in *C. gloeosporioides* (20) and *A. alternata* (21). These results suggest that YAP1 homologs have different functional roles in fungal development in different fungi.

Disruption of *PeAP1* in *P. expansum* did not affect spore germination and mycelial growth during the early stages of culture on either PDA or CYA media, but the colonies of the deletion mutants nearly stopped expanding after 3 days of culture and exhibited hyphal deformities during the later stages of culture (Fig. 1C and D, Fig. 3A through C; Fig. S3). Our results indicate that the accumulation of intracellular H_2_O_2_ in Δ*PeAP1* mutants may be responsible for the growth defects observed during the later stages of culture based on the following evidence: (i) Δ*PeAP1* mutants have higher levels of H_2_O_2_ than the WT strain after 5 days of culture (Fig. 4A and B); (ii) exogenous GSH partially restores the impaired growth of the mutants (Fig. 4C and D); and (iii) *PeAP1* deletion results in lower levels of antioxidant enzyme activity and GSH content (Fig. 4E and F; Fig. S4). Taken together, these findings highlight the important role that PeAP1 plays in intracellular H_2_O_2_ homeostasis during the normal vegetative growth of *P. expansum*.

### PeAP1 prevents the accumulation of host-derived H_2_O_2_ during biotrophic growth

Fungal pathogens must be able to detoxify or eliminate ROS-mediated host defense barriers in order to infect and grow in host tissues, and this potentially involves YAP1 homologs (43). In *U. maydis* (16) and *M. oryzae* (19), as well as in other plant pathogens, disruption of YAP1 homologs leads to reduced tolerance to oxidative stress and a decrease in virulence. In *C. heterostrophus* (22), *B. cinerea* (23), and *Fusarium graminearum* (44), YAP1 proteins mediate the response to H_2_O_2_ but do not affect virulence. In the present study, we observed that deletion of *PeAP1* results in increased sensitivity to H_2_O_2_ and a significant decrease in the virulence of the mutants on apple and pear fruits (Fig. 1 and Fig. 5A through C). These results suggest that the role of YAP1 homologs is conserved in oxidative stress response but not in virulence.

ROS generation is a hallmark of successful recognition of infection and the activation of plant defense response (45, 46). H_2_O_2_ accumulation in *M. oryzae* was detected at the plant site inoculated with *MoAP1* deletion mutants but not with WT strains, suggesting that MoAP1 prevents the accumulation of H_2_O_2_ during biotrophic growth (19). The NADPH oxidase inhibitor DPI can prevent H_2_O_2_ accumulation at infection sites inoculated with *U. maydis* and can restore the virulence of Δ*yap1* mutants (16). Our study also demonstrated that the loss of *PeAP1* in *P. expansum* resulted in increased H_2_O_2_ accumulation at the inoculation site in fruits and that inoculation of fruit with both the *PeAP1* mutant and DPI partially restored fungal virulence (Fig. 5D through F; Fig. S5). These findings indicate that PeAP1 participates in the detoxification of host-produced ROS and that excessive H_2_O_2_ levels contribute to the impaired pathogenicity of *PeAP1* mutants.

### Four novel PeAP1 target genes are involved in the oxidative stress response and/or virulence

The identification of H_2_O_2_-induced PeAP1 target genes by RNA sequencing analysis revealed similar functional categories of genes that are regulated by Yap1 in *U. maydis* (16) and MoAP1 in *M. oryzae* (19). Among these genes were some well-studied ones directly related to pathogenicity and novel ones, such as two peroxiredoxin genes (*PePrx1* and *PePrx2*), a glutathione S-transferase gene (*PeGST1*), and a thioredoxin gene (*PeTRX2*), that are likely to play a role in H_2_O_2_ resistance. In fungi, peroxiredoxin, glutathione S-transferase, and thioredoxin are considered significant enzymes that aid in neutralizing host-generated ROS during plant-microbe interactions (47 – 49). Recent studies have shown that the reduced expression of these enzymes likely causes a decrease in the ability to scavenge ROS originating from the host, ultimately leading to a reduction in virulence (47 – 49). In *P. expansum*, *PePrx1*, *PePrx2*, *PeGST1*, and *PeTRX2* contain YAP1 binding sites in their 1.5-kb promoter regions, and knockout mutants confirmed their involvement in virulence. Moreover, *PePrx1*, *PePrx2*, and *PeTRX2* were shown to be required for ameliorating oxidative stress in *P. expansum* (Fig. 9). The phenotypes of the knockout mutants were consistent with the phenotypes observed in the *PeAP1* mutants, suggesting that the PeAP1 regulator has a direct role in regulating antioxidant-related genes to respond to oxidative stress and maintain normal mycelial growth and virulence. Deletion of PeAP1 may also alter the transcript levels of many genes related to sulfur metabolism, including two well-studied virulence factors, *PesB* (30) and *PeSat* (31), that affect pathogenicity (Fig. 6C; Fig. S8). The relationship between PeAP1, sulfur metabolism, and fungal pathogenesis requires further investigation.

**FIG 10:**
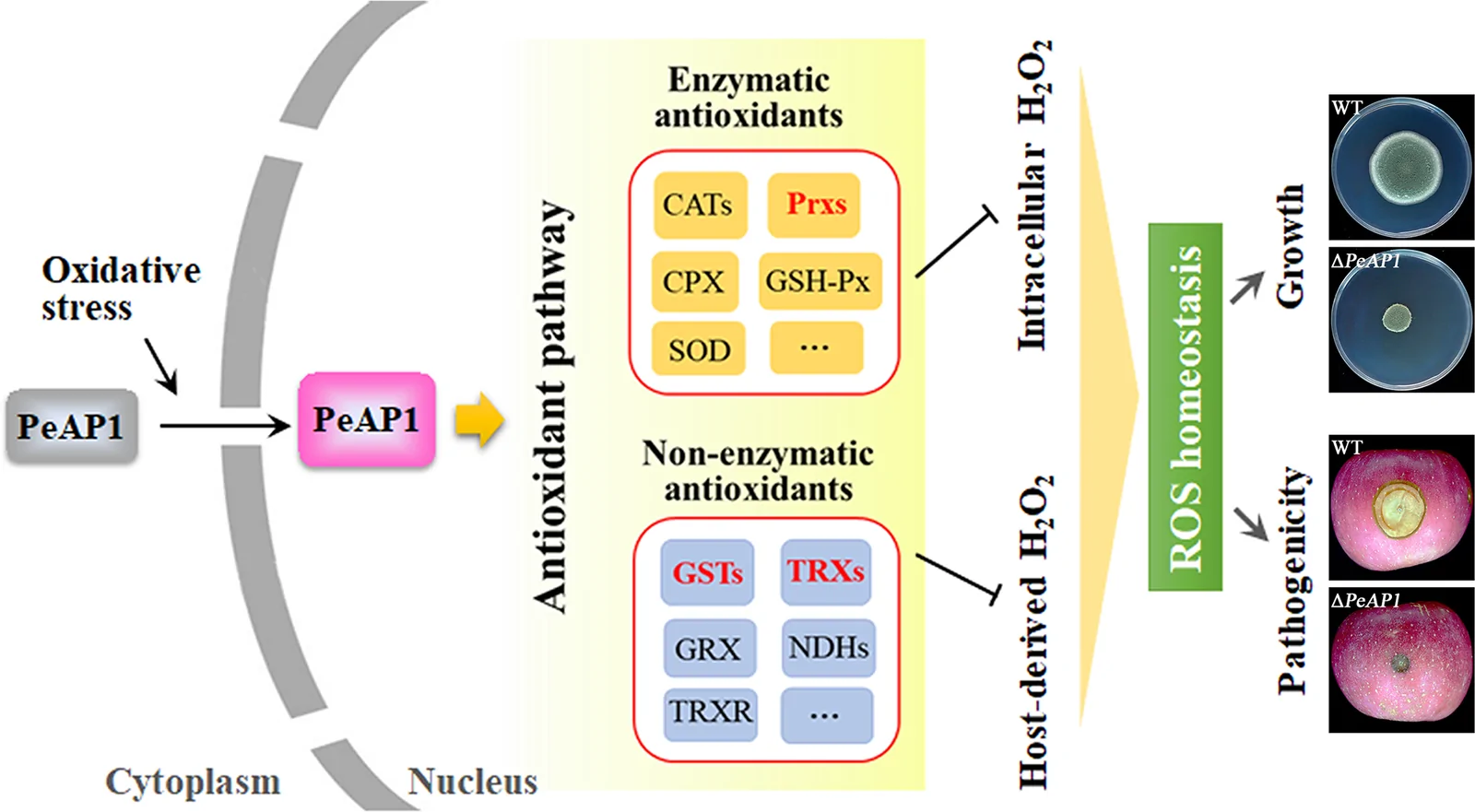
Proposed model for PeAP1 regulating the growth and pathogenicity of *P. expansum*. In response to oxidative stress, PeAP1 relocalizes from the cytoplasm to the nucleus, where it activates the expression of many genes involved in ROS detoxification and antioxidant biosynthesis. The activation of antioxidant pathway can efficiently eliminate both intracellular H_2_O_2_ during vegetative growth and host-derived H_2_O_2_ during biotrophic growth, maintaining intracellular and extracellular redox balance, thus ensuring the normal growth and pathogenicity of *P. expansum*.

In conclusion, our results provide evidence that the bZIP transcription factor PeAP1 acts as a central regulator of the oxidative stress response in *P. expansum*, and that there is a major link between PeAP1-mediated oxidative stress response and fungal growth and virulence (Fig. 10). RNA sequencing analysis revealed a number of H_2_O_2_-induced PeAP1 target genes, including four novel ones, *PePrx1*, *PePrx2*, *PeGST1*, and *PeTRX2*, whose functions were linked to PeAP1 and pathogenicity. These findings can be used to foster the development of efficient strategies to control blue mold in harvested fruits.

## MATERIALS AND METHODS

### Fungal strains and growth conditions

WT *P. expansum* strain T01, isolated from an infected apple fruit, was used as the source strain for the construction of gene deletion mutants (50). Both WT and mutant strains were grown on potato dextrose agar media at 25°C for 7 days to generate and collect fungal spores for the experiments. Liquid culture of fungal spores was conducted in CY extract media at 25°C on a rotary shaker set at 180 rpm.

### Sequence and cluster analysis of PeAP1

Protein sequences of PeAP1 and YAP1 homologs were downloaded from fungal genome databases (accession numbers: *P. expansum*, GCA_001008385.1; *Neurospora crassa*, GCA_000182925.2 and the relevant published literature (19 – 24, 38, 42, 44, 51). Multiple sequence alignments were conducted using BioEdit version 7.0 software. Maximum likelihood phylogenetic trees were generated with full-length protein sequences using MEGA version 5.0 software (52) with default settings and 1,000 bootstrap replications.

### Construction of gene knockout and complementation strains

Construction of gene deletion mutants of *PeAP1* and 12 putative PeAP1 target genes was conducted using a homologous recombination strategy and an *Agrobacterium*-mediated transformation (ATMT) method as described by Li et al. (50). Briefly, the 5′- and 3′-flanking regions of the targeted knockout genes were amplified from *P. expansum* DNA. The products were purified and inserted into the upstream or downstream of the hygromycin B resistance cassette (*hph*) in the pCHPH vector to yield the final gene knockout vectors pCHPH-targeted gene. Hygromycin B (250 µg/mL) was used to select transformants during transformation. Positive transformants for each target gene were verified by PCR and further confirmed by Southern blot analysis using an *hph*-specific probe (50). To generate double-deletion strains, the *hph* gene of pCHPH vector was replaced with a neomycin resistance (*neo*) gene to produce the vector, pCNEO. *PePrx1&*2 and *PeGST1&*5 double mutant strains were generated by transferring pCNEO-*PePrx2* and pCNEO-*PeGST5* deletion constructs into Δ*PePrx1* and Δ*PeGST1* via ATMT, respectively. The full ORF sequence of each targeted gene with its promoter and terminator was cloned into pCNEO for use in generating gene complementation strains. Both the double-deletion strains and the complementary strains were screened using 250-µg/mL G418, then confirmed by PCR and assessed for phenotype complementation. Primers used for each gene knockout and complementation in this study are listed in Table S1 and S4.

### Phenotype analysis

Fungal phenotypes were characterized according to the methods of Chen et al. (31). Three biological replicates were used in all of the assessments.

#### Mycelial growth

The mycelial growth of WT, target gene deletion mutants, and the corresponding complementary strains, under the different concentration of H_2_O_2_ and/or GSH were evaluated. Briefly, 5 µL of a conidial suspension was adjusted to 10^5^ conidia/mL and centrally spotted onto PDA media supplemented with 0, 2.5, or 5.0-M H_2_O_2_ or GSH and then incubated in the dark at 25°C for 7 days. Mycelial growth was assessed by measuring two perpendicular diameters of each colony daily. Four plates were used per strain.

#### Conidiation

Conidia of the tested *P. expansum* strains were collected by flooding a PDA plate with 0.05% Tween 20 after 10 days of incubation. The collected solution was then filtered through one layer of miracloth, and the number of conidia in the collected filtrate was counted using an automated cell counter. Four plates of each strain were assessed.

#### Conidial germination

Conidia suspensions (5 × 10^7^ conidia/mL) of the *P. expansum* strains were spread on a cellophane sheet placed on PDA media with or without the addition of 2.5-mM H_2_O_2_. The percentage of germinated conidia of each suspension was assessed using light microscopy after 5–12 h of incubation at 25°C.

#### Pathogenicity

Apple and pear fruits and grape berries were used in the pathogenicity assay. Fruits were wounded (3 mm wide and 3 mm deep) with a sterile nail at the equator of each fruit, and then each wound was inoculated with 5 µL of a spore suspension (10^5^ conidia/mL). Inoculated fruit were stored at 25°C for 7 days, and the diameter of fruit lesion was recorded daily. For the DPI treatment, a final concentration of 10-µM DPI was added directly to the conidial suspension of each of the *P. expansum* strains prior to inoculation (16). A dissolved solution of DPI [0.1% (vol/vol) DMSO] was used as a control. Twenty fruits were inoculated per strain.

### Gene expression, protein abundance, and subcellular localization of PeAP1 in response to H_2_O_2_

The WT strain was grown in CY media for 24 h and then shifted to media amended with either 0-, 1.0-, 2.5-, 5.0-, or 10.0-mM H_2_O_2_ and incubated for an additional 1 h to assess the expression of *PeAP1* in response to oxidative stress conditions. To investigate the transcription of *PeAP1* in response to short-term treatments of H_2_O_2_, overnight-grown mycelia of the WT strain were shifted to media containing 5-mM H_2_O_2_ and then incubated for another 5, 15, 30, 60, 120, and 240 min. The mycelia were then harvested and immediately frozen. Total RNA extraction and RT-qPCR were carried out as previously described (50) using the primer pair, qPeAP1-F/R (Table S1).

A C-terminal *PeAP1::eGFP* fusion gene under the control of endogenous *PeAP1* promoter was constructed to assess PeAP1 protein abundance under oxidative conditions. A genomic fragment containing the coding region of *PeAP1* gene and its endogenous promoter region was amplified using the primers gAP1-F/R (Table S1) and inserted into the 5-end of eGFP in the binary vector pCNEO-eGFP (31) to yield the fusion expression vector PeAP1-GFP. The constructed vector was transformed into Δ*PeAP1* mutant using the ATMT method. Transformants were selected on CY media containing 250-µg/mL G418 and assessed for H_2_O_2_ sensitivity and green fluorescence. Shift experiments with the positive transformant Δ*PeAP1/PeAP1::eGFP* were performed as described above. The collected samples of mycelia were used for protein extraction and Western blot analysis as described by Chen et al. (30). PeAP1::eGFP and histone H3 were detected using anti-GFP mouse monoclonal antibody 7G9 (M20004M, Abmart) at 1:5,000 and the mouse anti-histone H3 monoclonal antibody 3B9 (M20008, Abmart) at 1:5,000, respectively.

For subcellular localization of PeAP1, spores of Δ*PeAP1/PeAP1::eGFP* were incubated in CY media at 25°C for 14 h, and then the germinated spores were shifted to CY media with or without 5-mM H_2_O_2_ for another 2 h. Fluorescent signals were examined with a Leica TCS SP5 confocal laser-scanning microscope. DAPI (Sigma-Aldrich, Saint Louis, MO, USA) was used for the staining of nuclei according to the manufacturer’s instructions.

### H_2_O_2_ detection

DAB staining solution (0.5 mg/mL) was prepared by mixing DAB powder in 0.2-M Na_2_HPO_4_-0.1-M citric acid buffer (pH 3.8) and was used to determine the accumulation of intracellular H_2_O_2_ in the different *P. expansum* strains (36). Fresh mycelia (25 mg) were collected after the different strains were cultured for 3, 5, and 7 days on PDA. The collected mycelia were then immersed in 1-mL DAB staining solution for 12 h in darkness. The mycelia were then filtered used to examine the color change of the staining solution and the mycelia.

Intracellular H_2_O_2_ content in the different *P. expansum* strains was determined using a commercial assay kit (Solarbio Life Science, Beijing, China). Conidial suspensions (10^5^ conidia/mL) were spotted onto PDA and incubated in the dark at 25°C for 7 days. Mycelia were harvested at 3, 5, and 7 dpi and used to assess the level of H_2_O_2_. H_2_O_2_ accumulation around inoculation sites in apple fruits was also assessed. Conidial suspensions of WT and Δ*PeAP1* mutant strains were administered into the wound site in apple fruits, and identical quantities of host tissues were taken from the disease-health junction at 1, 3, and 5 dpi. H_2_O_2_ content was measured by measuring absorbance at 240 nm.

### Measurement of GSH content and antioxidant enzyme activity

The mycelia used for GSH, GR, CAT, and POD analyses were prepared and collected in the same way the mycelia used for *in vitro* H_2_O_2_ detection. Quantifications of GSH and the assay for GR, CAT, and POD activity were conducted using commercial assay kits (Solarbio Life Science, Beijing, China) following the manufacturer’s instructions. GSH content was determined by measuring absorbance at 412 nm. One GR unit was calculated as the quantity of enzyme required to cause a decrease of 0.01 absorbance at 340 nm/min. CAT activity was determined by recording the decrease in absorbance at 240 nm due to H_2_O_2_ consumption. POD activity was measured at 470 nm within 1 min. The experiments were repeated three times.

### RNA-seq analysis

WT and Δ*PeAP1* strains were inoculated in CY media at 25°C and grown for 24 h on a rotary shaker set at 180 rpm. The cultures were then divided and one-half of the culture media was supplemented with 5-mM H_2_O_2_ and incubated for another 1 h. Mycelia were then separately harvested from both halves of media using one layer of miracloth, washed three times with distilled water, and then frozen in liquid nitrogen. Material derived from three independent experiments was used for RNA isolation (TRIzol procedure). cDNA library construction and Illumina sequencing were performed according to the method reported by Li et al. (53). Genes with a log2 fold change of ≥2.0 and a false discovery rate of <0.05 were considered to be differentially expressed. Gene Ontology function prediction and KEGG pathway analysis of DEGs were conducted as described by Sun et al. (54). RNA-seq results were verified by RT-qPCR analysis of 20 H_2_O_2_-induced PeAP1 target genes.

### Statistical analysis

Quantitative data presented in this study are the mean ± standard error of the mean. Data were statistically anlyzed by a one-way analysis of variance using SPSS version 11.5 (Chicago, IL, USA). Statistical significance of the data were assessed using Student’s *t*-test or Duncan’s test. Differences at *P* < 0.05 were considered significant.

## Data Availability

The raw sequences obtained have been deposited in the NCBI Sequence Read Archive under the accession number PRJNA944696.

## SUPPLEMENTAL MATERIAL

Supplemental figures and tables spectrum.03808-22-s0001.pdf Figure S1 to S8; Tables S1 to S4.

ASM does not own the copyrights to Supplemental Material that may be linked to, or accessed through, an article. The authors have granted ASM a non-exclusive, world-wide license to publish the Supplemental Material files. Please contact the corresponding author directly for reuse.
