# Supplementary material for: PeAP1-mediated oxidative stress response plays an important role in the growth and pathogenicity of Penicillium expansum
Source: Microbiol Spectr. 2023 Sep 21;11(5):e03808-22. doi: 10.1128/spectrum.03808-22 (PMC10581040; doi:10.1128/spectrum.03808-22)
Supplement: Supplemental figures and tables — Figure S1 to S8; Tables S1 to S4. [file spectrum.03808-22-s0001.pdf]

PeAP1-mediated oxidative stress response plays an important role in the growth and pathogenicity of *Penicillium expansum*

Yong Chen[1,2], Yichen Zhang[1,3], Dongying Xu[1,3], Zhanquan Zhang[1,2], Boqiang Li[1,2], Shiping Tian[1,2,3]

[1]State Key Laboratory of Plant Diversity and Specialty Crops, Institute of Botany, Chinese Academy of Sciences, Beijing, China

[2]China National Botanical Garden, Beijing, China

[3]University of Chinese Academy of Sciences, Beijing, China

Correspondence to: Shiping Tian; tsp@ibcas.ac.cn; Boqiang Li; bqli@ibcas.ac.cn

SUPPLEMENTAL FIGURES

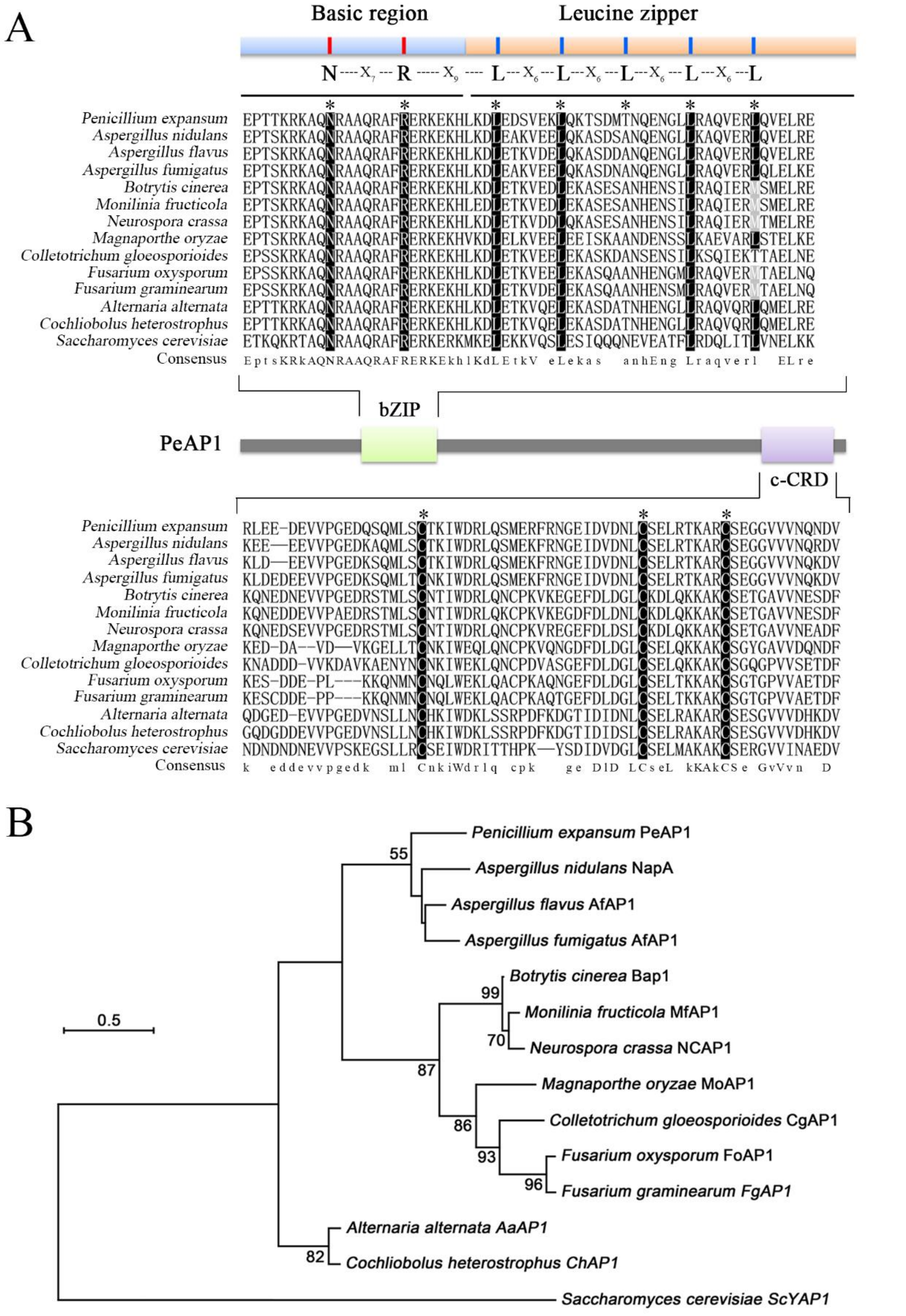

FIG S1 Functional domains and phylogenetic analysis of PeAP1. (A) Alignment

of the conserved bZIP and c-CRD domains of YAP1-like proteins in yeast and several important pathogenic fungi: *P. expansum* (PEG01534), *Aspergillus nidulans* (Q5AW17), *A. flavus* (UDD55270), *A. fumigatus* (EAL88844), *Botrytis cinereal* (ATZ56463), *Monilinia fructicola* (ALO75528), *Neurospora crassa* (CAB91681), *M. oryzae* (EDK00544), *C. gloeosporioides* (AOA33446), *Fusarium oxysporum* (AFN84618), *F. graminearum* (XP_011319920), *Alternaria alternata* (ACM50933), *C. heterostrophus* (AAS64313), *S. cerevisiae* (CAA41536). Asterisks indicate conserved amino acid residues. **(B)** Phylogenetic analysis of fungal YAP1-like proteins. The phylogenetic tree was constructed with Mega 5.0 software using a maximum likelihood (ML) method. The horizontal bar represents the relative distance in the phylogenetic tree.

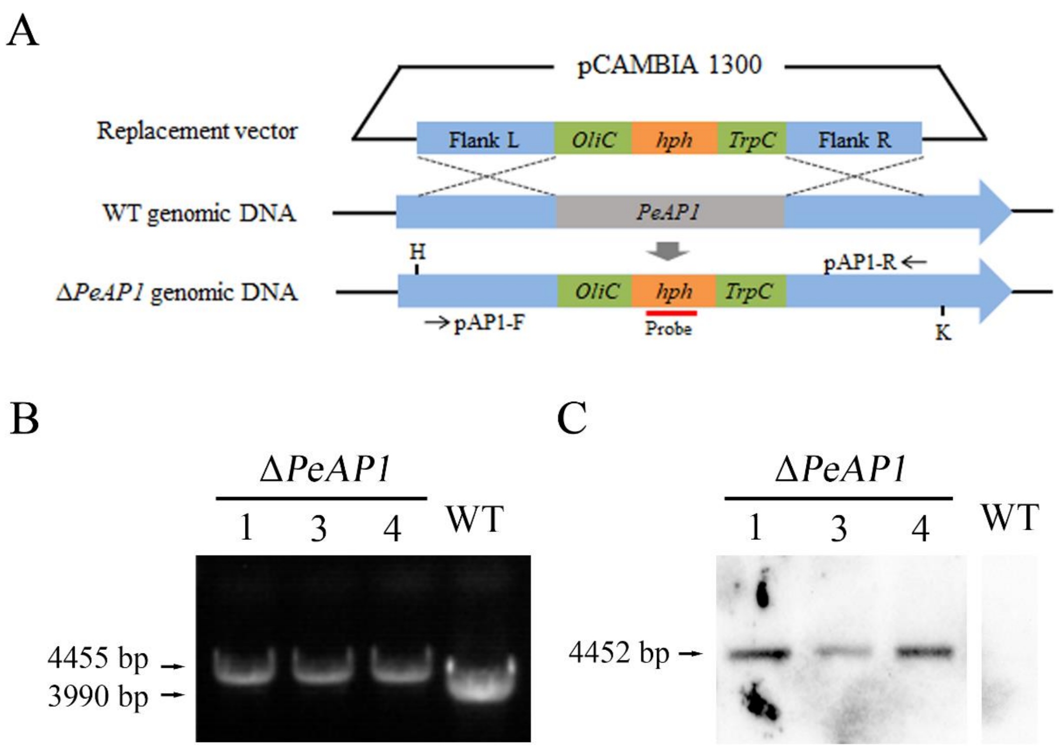

FIG S2 Targeted gene disruption of *PeAP1*. **(A)** Schematic illustration of the gene replacement strategy used for the replacement of PeAP1 with a hygromycin B resistance cassette. **(B)** Confirmation of the positive transformants by PCR using genomic DNA as a template. **(C)** Confirmation of positive transformants by Southern blot analysis. Genomic DNA was digested with *Hin*dIII and *Kpn*I and hybridized with an *hph*-specific probe.

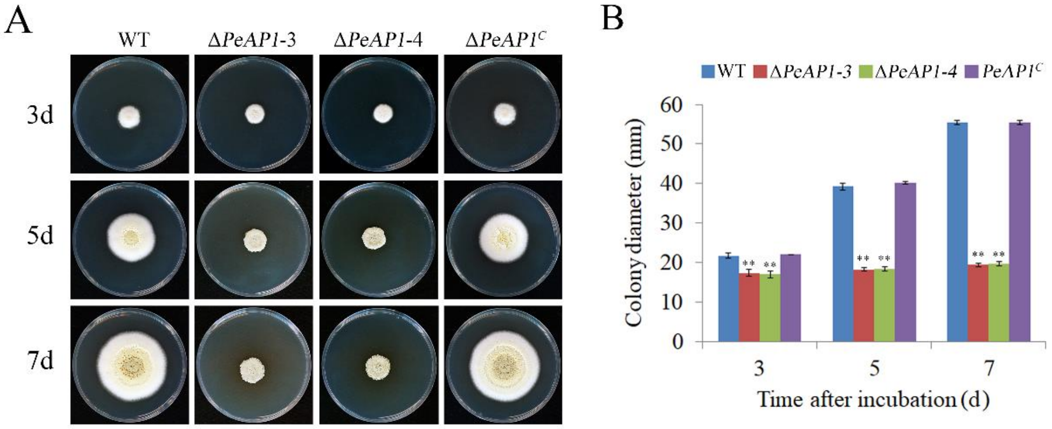

FIG S3 Loss of *PeAP1* affects hyphal development and extension in mutant strain grown on CYA media. WT, *PeAP1* mutant. and *PeMetR*^{C} strains of *P. expansum* were cultured on CYA media at 25°C for 10 d. Growth of the strains was photographically documented **(A)** and quantitatively analyzed **(B)**. Data represent the mean ± SEM (n = 3). ** indicates a significant difference at P < 0.01.

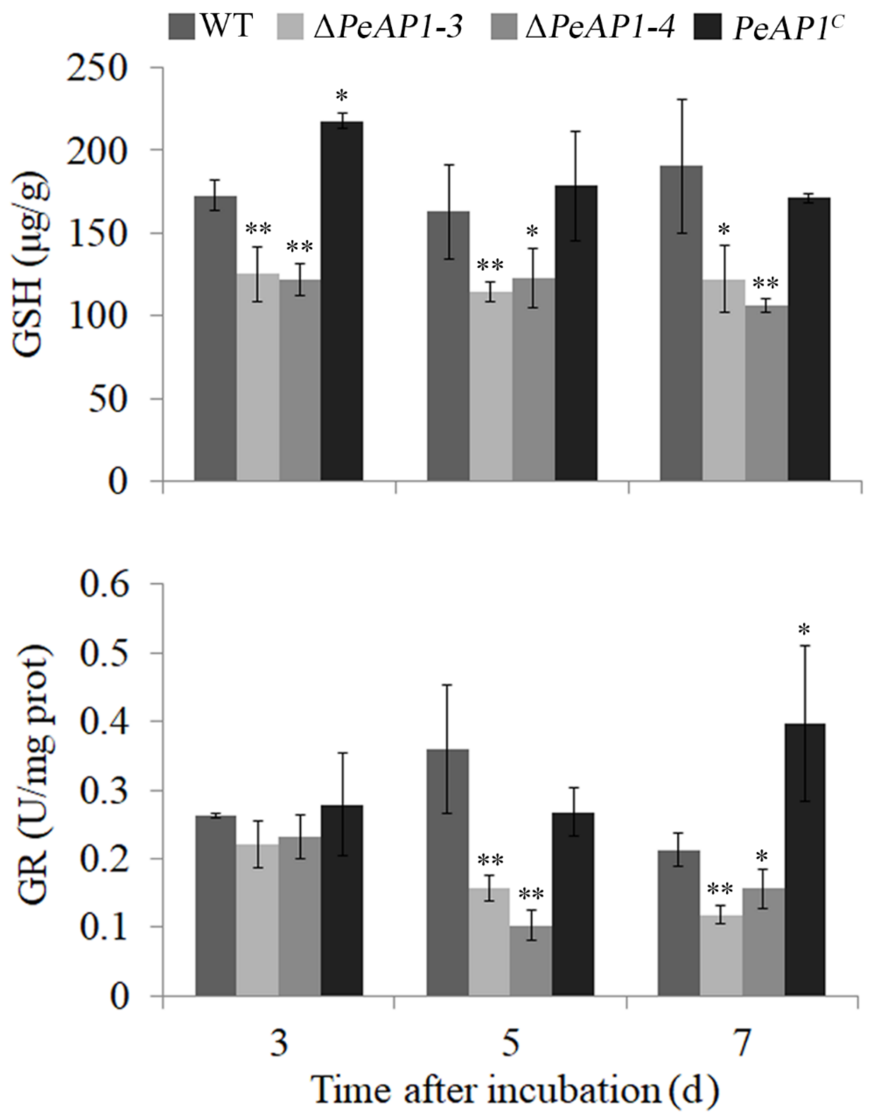

FIG S4 PeAP1 regulates the production of intracellular GSH. WT, *PeAP1* mutant, and *PeAP1*^{C} strains of *P. expansum* were cultured on PDA media at 25°C for 3, 5, and 7 d. Identical quantities of fresh mycelia of each of the tested strains were used for analysis of GSH content and glutathione reductase (GR) activity. Data represent the mean ± SEM (n = 3). * indicates a significant difference at $P < 0.05$; ** at $P < 0.01$.

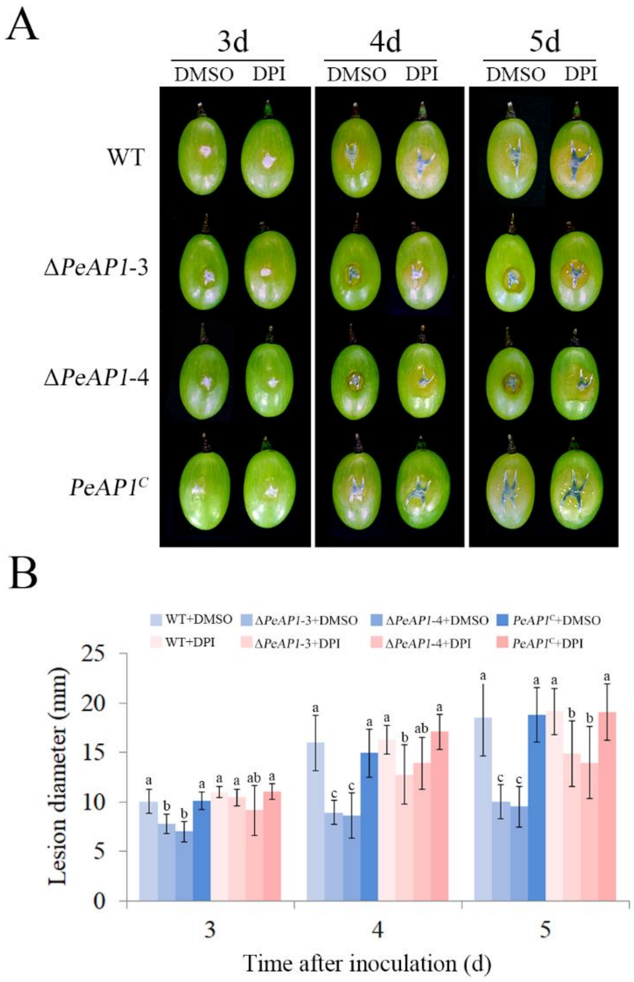

FIG S5 DPI partially restores the virulence of *PeAP1* mutants on grape fruit. Time course of decay development in grape berries inoculated with conidia of WT, *PeAP1* mutant, and *PeAP1*^{C} strains of *P. expansum* along with DMSO or DPI dissolved in DMSO. The infection process for each of the strains was photographically documented **(A)** and quantitatively analyzed at 3, 4 and 5 dpi **(B)**. Data represent the mean ± SEM (n = 3). Columns with different letters are significantly different from each other as determined by a least significant difference test ($P < 0.05$).

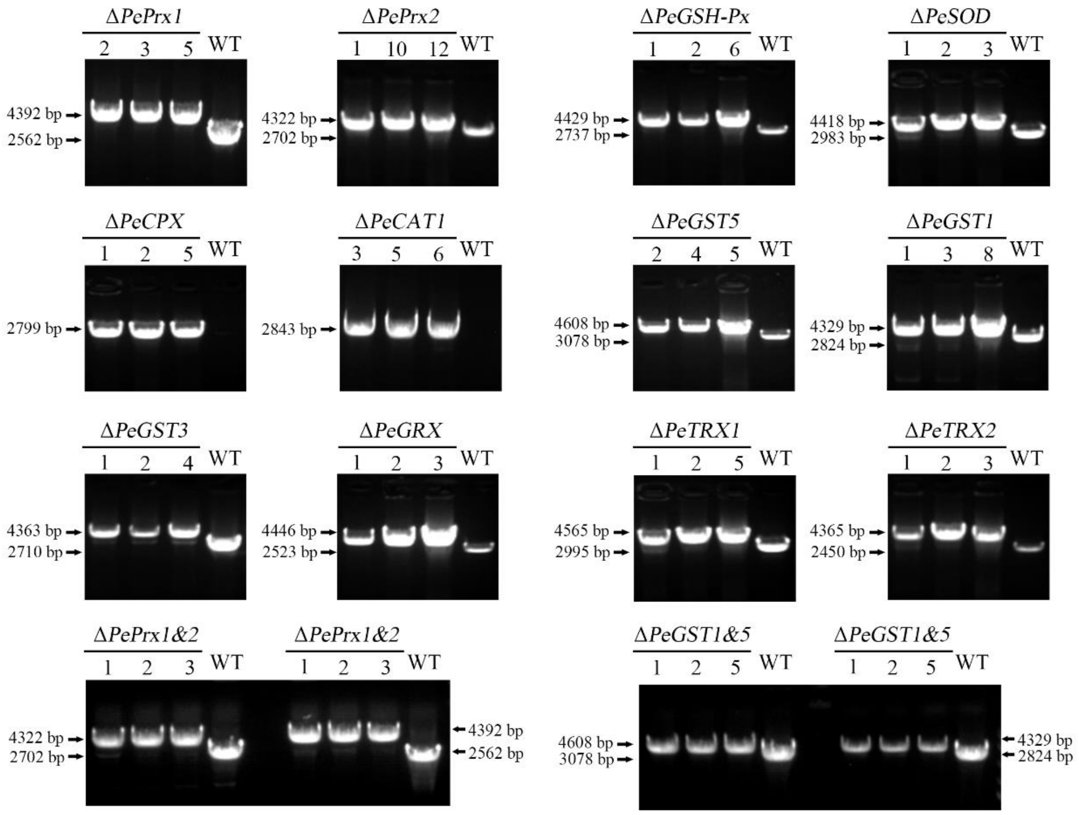

FIG S6 PCR verification of positive transformants of single-gene deletion mutants of *PePrx1*, *PePrx2*, *PeGSH-Px*, *PeSOD*, *PeCPX*, *PeCAT1*, *PeGST5*, *PeGST1*, *PeGST3*, *PEGRX*, *PeTRX1*, and *PeTRX2*, and double-gene deletion mutants of *PePrx1&2* and *PeGST1&5*. Primers used in the PCR analysis are listed in Table S4.

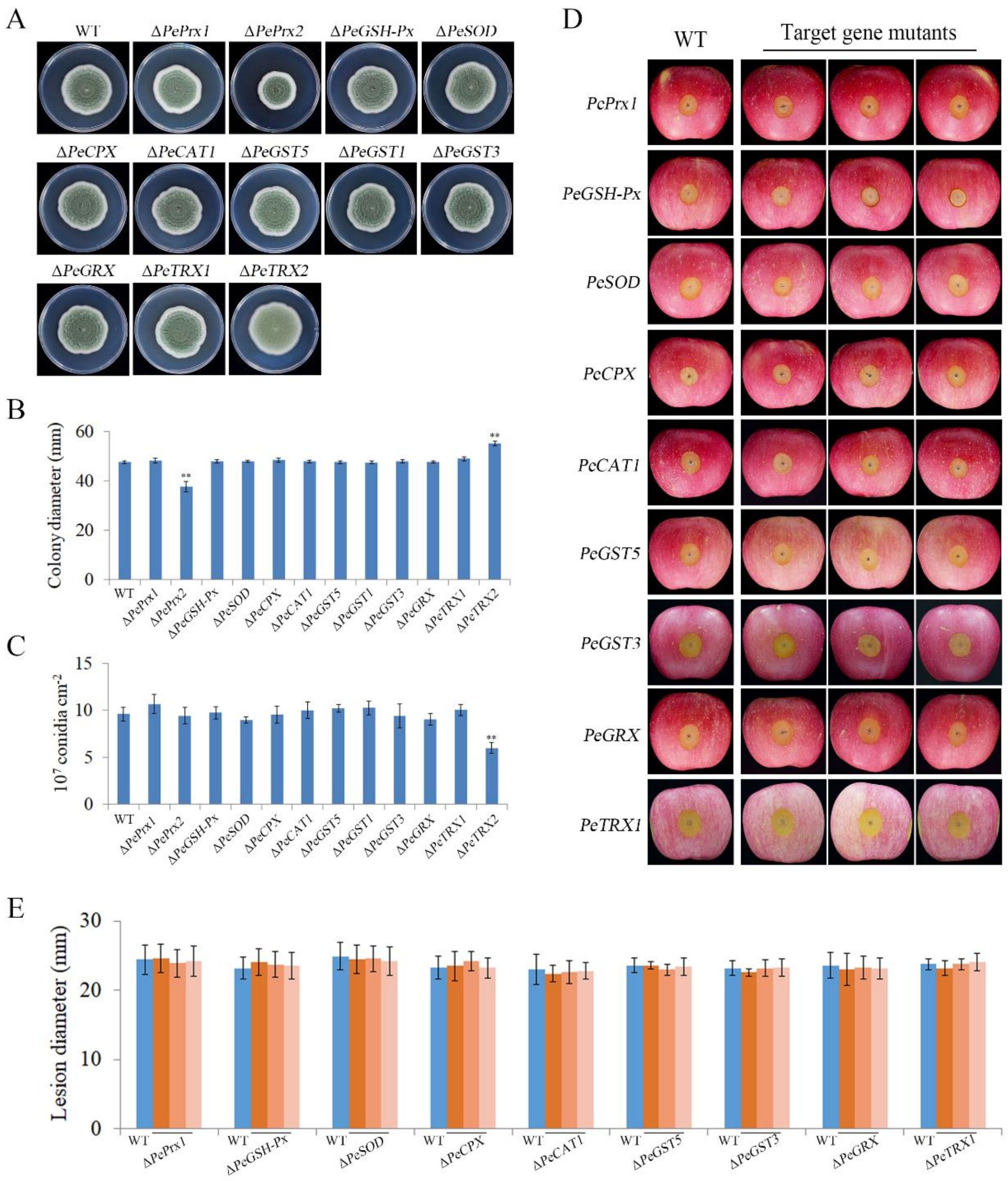

FIG S7 Phenotypic observations of single-gene disruptions of twelve PeAP1-regulated genes. (A-C) The tested strains were cultured on PDA media at 25°C for 10 d. Growth of the strains was photographically documented and quantitatively analyzed at 7 dpi. Conidiation in the different strains was quantitatively analyzed at 10 dpi. Data represent the mean ± SEM (n = 3). ** indicates a significant difference at P < 0.01. **(D, E)** Conidia of the tested strains were pipetted into wounds

made in apple fruit. Fruit were then incubated at 25 °C for 7d. Photographs were taken at 5 dpi. Lesion diameter was measured at 5 dpi. Data represent the mean ± SEM (n = 3).

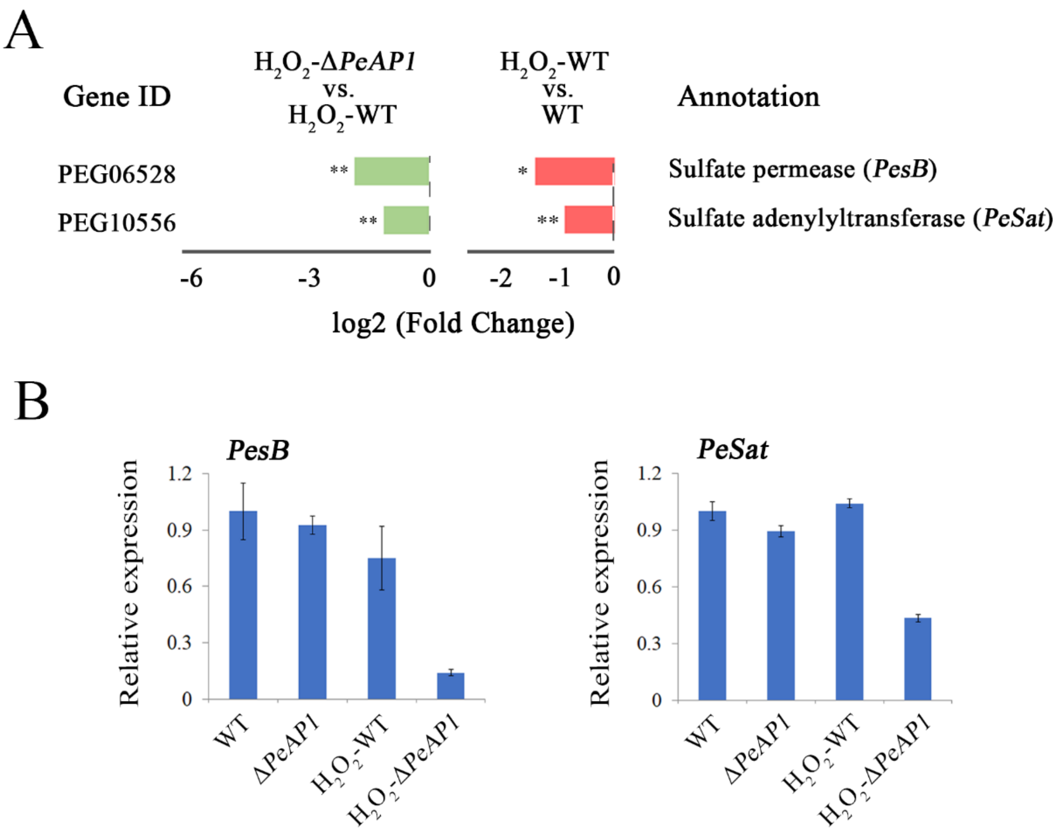

FIG S8 PeAP1 regulates the expression of two genes involved in sulfur metabolism. (A) Expression pattern of DEGs involved in virulence in the Δ*PeAP1* mutant and WT strains of *P. expansum* in response to an H_2O_2 treatment. *indicates a significant difference at $P < 0.05$; ** at $P < 0.01$. **(B)** Verification of the DEGs involved in virulence by RT-qPCR. Primers used are listed in Table S3. Relative expression was normalized using *β-tublin* as an internal control. Data represent the mean ± SEM (n = 3).

SUPPLEMENTAL TABLES

TABLE S1 Sequence of primer pairs used for manipulation of *PeAP1* in *P.expansum* and identification of mutant strains.

**Primers**	**Primer sequences (5' - 3')**	**Purpose**
qPeAP1-F/R	ACAGTCCTTCGTCTCGATCGA / GGAAGTAGCGCTGCTAGGAGTT	RT-qPCR analysis for *PeAP1*
Histone H3-F/R	AGATCCGTCGCTACCAGAAGTC / TTGAAGTCCTGGGCGATCTC	RT-qPCR analysis for *PeAP1*
PeAP1up-F/R	CAGATCGACCGAGATATGCATA / TGTGACGGTTTAACTTCCAATG	Cloning *PeAP1* Flank L (1085 bp)
PeAP1dn-F/R	TGGCTTGCTTCCAGGATGCT / GCCTTTTTGACTCTGATAGCGC	Cloning *PeAP1* Flank R (1003 bp)
PeAP1fl-F/R	CAGATCGACCGAGATATGCATA / GCCTTTTTGACTCTGATAGCG	Cloning full-length *PeAP1* (3990 bp) for complementation
pPeAP1-F/R	CAGATCGACCGAGATATGCATA / GCCTTTTTGACTCTGATAGCG	PCR detecting *PeAP1*
Hph-F/R	TCACCCCCATCTCAACTCCA / TGCTCCATACAAGCCAACCAC	Southern probe for *PeAP1*
gPeAP1-F/R	GCCATGGTGGATTCTGATTTAG / CTTGGCTCGTCCCATAATGG	Subcellular localization for PeAP1

TABLE S2 List of downregulated genes in the *PeAP1* mutant and upregulated in WT strains in response to H_2O_2.

Gene ID[a]	Annotation	Log2 (Fold change)		FPKM mean[b]	Putative PeAP1 binding sites[c]
		H_2O_2-Δ*PeAP1* vs H_2O_2-WT	H_2O_2-WT vs WT		
PEG10499	Glutathione-independent glyoxalase	-10.98	7.46	10.71	Yes
PEG08834	Aldehyde reductase	-9.67	4.85	140.77	Yes
PEG01141	NmrA-like family protein	-8.68	6.91	21.57	Yes
PEG03093	Uncharacterized protein	-7.98	2.78	42.59	Yes
PEG03317	Peroxiredoxin	-7.95	7.36	74.58	Yes
PEG05950	NAD-dependent epimerase	-7.60	6.80	88.20	Yes
PEG11490	Astacin	-7.44	7.11	2.18	No
PEG10498	Dimeric alpha-beta barrel	-7.31	7.41	30.17	Yes
PEG08064	Catalase-peroxidase	-6.94	5.95	259.61	Yes
PEG02184	NADH oxidase	-6.90	2.46	3.91	Yes
PEG09967	Uncharacterized protein	-6.49	5.95	205.18	No
PEG09232	Catalase	-6.37	4.28	319.23	Yes
PEG08105	Quinone oxidoreductase	-6.36	4.68	54.84	Yes
PEG08383	Glutathione transferase 3	-5.98	2.43	387.41	Yes
PEG09227	Cupin domain protein	-5.91	6.26	71.41	No
PEG04373	Nitrilotriacetate monooxygenase component A	-5.77	2.89	3.98	No
PEG02965	Short chain dehydrogenase	-5.39	1.07	7.03	Yes
PEG05915	NmrA-like family protein	-5.35	3.00	273.57	Yes
PEG08936	Isoflavone reductase family protein	-5.30	5.49	261.36	Yes
PEG07117	SnoaL-like domain	-5.19	3.39	127.44	Yes
PEG00695	Isochorismatase hydrolase	-4.90	2.37	20.48	Yes
PEG02421	Thioredoxin	-4.79	1.82	63.67	Yes
PEG01294	N-ethylmaleimide reductase	-4.75	1.85	5.09	Yes
PEG05285	Glutathione S-transferase	-4.72	9.08	19.99	Yes
PEG10161	Short chain dehydrogenase	-4.68	5.87	71.90	Yes
PEG05951	Zinc-binding dehydrogenase	-4.61	4.45	40.69	Yes
PEG07812	Isoflavone reductase	-4.25	4.04	33.60	No
PEG10835	NADH oxidase	-4.22	1.02	5.80	Yes
PEG08126	Glutathione S-transferase	-4.17	4.14	354.13	Yes
PEG02146	Short-chain dehydrogenase	-4.13	2.49	16.75	No
PEG00958	Flavin reductase	-4.08	2.63	46.16	Yes
PEG06993	Catalase	-3.87	2.36	3.25	Yes
PEG08141	NmrA-like family protein	-3.87	3.86	27.17	Yes
PEG11418	Lysine-ketoglutarate reductase	-3.85	4.93	0.59	Yes
PEG06482	Quinone oxidoreductas	-3.85	1.95	82.09	Yes
PEG07371	Oxalocrotonate tautomerase	-3.74	3.43	1.16	Yes

PEG01820	HypA-like protein	-3.72	4.74	224.80	Yes
PEG07163	Uncharacterized protein	-3.62	1.22	56.50	No
PEG08947	Glutaredoxin Grx1	-3.55	3.46	586.80	Yes
PEG02796	Uncharacterized protein	-3.51	2.99	9.33	Yes
PEG07370	NAD dependent epimerase/dehydratase	-3.46	3.54	69.00	Yes
PEG05529	Thioredoxin	-3.41	2.79	397.41	Yes
PEG10213	NADP-dependent hydroxydehydrogenase	-3.38	1.17	52.15	Yes
PEG10402	Polyketide synthase	-3.02	3.56	22.70	Yes
PEG07635	Uncharacterized protein	-2.85	3.06	1.41	Yes
PEG05619	Uncharacterized protein	-2.80	1.56	67.83	No
PEG07596	Oxidoreductase	-2.79	1.26	33.20	Yes
PEG04883	Glutathione S-transferase	-2.60	1.93	5.90	Yes
PEG10976	NAD dependent epimerase	-2.56	1.55	0.60	No
PEG03029	Eukaryotic aspartyl protease	-2.49	1.86	0.04	No
PEG08479	Glutathione peroxidase	-2.40	4.05	217.55	No
PEG11761	NADH oxidase family	-2.40	1.06	35.43	Yes
PEG09751	HD domain-containing protein	-2.39	6.08	25.25	Yes
PEG11626	Aflatoxin b1 aldehyde reductase-like protein	-2.31	1.37	35.36	Yes
PEG06011	GMC oxidoreductase	-2.27	1.63	34.63	Yes
PEG09485	Glutathione reductase	-2.24	1.32	44.52	No
PEG06933	Uncharacterized protein	-2.21	1.51	124.27	Yes
PEG05286	C6 finger domain protein	-2.18	4.42	165.24	No
PEG02047	Glutathione-dependent formaldehyde-activating enzyme	-2.13	1.07	4.51	Yes
PEG11087	Predicted acyl esterases	-2.12	2.70	0.97	Yes
PEG05699	Ethyl tert-butyl ether degradation EthD	-2.11	1.07	238.90	Yes
PEG07945	Pirin domain protein	-2.11	1.55	12.60	Yes
PEG00993	Transaldolase	-2.01	2.86	1.64	No
PEG08441	Glutathione S-transferase	-1.98	3.04	38.22	Yes
PEG04204	Thioredoxin reductase	-1.94	1.71	40.64	No
PEG00026	Uncharacterized protein	-1.92	3.09	4.50	No
PEG05350	Peroxiredoxin	-1.92	2.22	398.78	Yes
PEG00369	2,4-dienoyl-CoA reductase	-1.88	1.11	0.25	Yes
PEG01980	Ribosome biogenesis GTPase Lsg1	-1.86	5.35	0.21	Yes
PEG05033	ABC multidrug transporter SitT	-1.85	5.37	13.75	Yes
PEG02801	Amino acid permease	-1.84	1.24	2.07	No
PEG09555	2OG-Fe (II) oxygenase superfamily	-1.82	1.06	0.53	Yes
PEG09738	Glutathione S-transferase	-1.79	1.19	8.19	Yes
PEG05022	Polygalacturonase	-1.71	1.41	5.80	No
PEG09752	Alpha/beta hydrolase family	-1.64	5.61	1.09	Yes
PEG08915	Aldehyde reductase, putative	-1.62	2.71	7.84	Yes
PEG09736	Uncharacterized protein	-1.62	1.78	0.94	Yes
PEG04268	3,4-dihydroxy-2-butanone 4-phosphate synthase	-1.61	1.60	153.04	No

PEG00852	Proline utilization trans-activator	-1.56	1.26	0.30	Yes
PEG05442	Oleate delta-12 desaturase	-1.51	1.17	20.83	No
PEG09733	Long-chain-fatty-acid-CoA ligase	-1.48	5.01	6.58	Yes
PEG07109	L-ornithine N5 monooxygenase	-1.42	1.19	40.61	Yes
PEG06744	Nucleolar GTPase	-1.40	1.84	78.90	No
PEG09734	Pyridine nucleotide-disulfide oxidoreductase	-1.38	4.92	4.07	No
PEG01817	NADH pyrophosphatase	-1.38	1.03	4.65	No
PEG03474	Ketoreductase	-1.38	1.39	68.98	No
PEG08619	60S ribosome biogenesis protein Rrp14	-1.37	2.20	8.79	Yes
PEG10241	Alcohol dehydrogenase	-1.36	2.00	62.09	No
PEG07048	Short chain dehydrogenase	-1.35	1.73	7.99	Yes
PEG01528	Apoptosis-antagonizing transcription factor	-1.34	1.57	14.11	Yes
PEG09270	Gar1/Naf1 RNA binding region	-1.32	1.43	6.10	Yes
PEG09288	Arylsulfotransferase	-1.31	3.31	1.19	Yes
PEG08371	RTA1 like protein	-1.30	1.04	0.15	Yes
PEG04985	Superoxide dismutase	-1.29	1.63	48.65	No
PEG05485	Uncharacterized protein	-1.29	2.04	2.44	No
PEG03559	MFS monocarboxylate transporter	-1.27	1.44	0.65	Yes
PEG05034	HC-toxin synthetase	-1.26	4.05	1.65	No
PEG10539	RNA binding protein	-1.25	1.04	15.52	Yes
PEG11488	Thiolase	-1.25	1.00	18.62	Yes
PEG00237	Calcineurin-like phosphoesterase	-1.25	1.22	1.32	No
PEG09729	FRE family ferric-chelate reductase	-1.23	3.66	1.85	Yes
PEG05209	Uncharacterized protein	-1.22	1.62	69.71	No
PEG06420	ATP-dependent RNA helicase	-1.21	1.63	5.88	No
PEG06807	Pescadillo N-terminus	-1.21	1.01	26.93	Yes
PEG04125	Thiol methyltransferase	-1.20	1.72	12.07	No
PEG09019	Brix domain	-1.19	1.65	26.82	Yes
PEG03764	Amino acid transporter	-1.19	1.83	3.91	No
PEG03381	Pre-rRNA processing protein Tsr1	-1.18	1.15	15.29	No
PEG05032	Acetyltransferase SidF	-1.17	2.16	2.86	Yes
PEG09788	Uncharacterized protein	-1.17	1.07	22.90	No
PEG01598	Nucleolar RNA methyltransferase	-1.16	1.16	24.95	No
PEG00977	Proteasome maturation ans ribosome synthesis protein	-1.16	1.31	16.53	No
PEG02737	Methyltransferase	-1.15	1.90	3.15	Yes
PEG07748	WD repeat protein	-1.14	1.61	18.83	No
PEG03648	MFS multidrug transporter	-1.13	1.16	4.29	Yes
PEG08280	RNA processing protein Grc3	-1.13	1.37	7.47	Yes
PEG00149	RNA polymerase I-specific transcription-initiation factor	-1.13	1.37	4.41	No
PEG04440	U3 small nucleolar ribonucleoprotein Lcp5	-1.13	1.40	26.44	No
PEG04912	Uncharacterized protein	-1.12	1.38	26.50	No
PEG05551	Quinone oxidoreductase	-1.11	2.48	53.48	Yes

PEG09018	ATP-dependent RNA helicase	-1.11	1.03	18.56	Yes
PEG01276	MFS maltose permease	-1.10	1.93	1.19	No
PEG01537	Importin beta-4 subunit	-1.10	1.18	75.04	No
PEG09941	rRNA processing protein	-1.09	1.37	19.87	Yes
PEG04640	U3 small nucleolar ribonucleoprotein	-1.09	1.44	16.19	Yes
PEG00617	Nucleolar protein 9	-1.08	1.32	18.06	Yes
PEG02802	DEAD box RNA helicase	-1.07	1.42	11.65	Yes
PEG04900	Small nucleolar ribonucleoprotein complex subunit	-1.07	1.36	19.34	Yes
PEG03771	Dip2/Utp12 Family	-1.07	1.36	26.87	Yes
PEG02091	Aldo/keto reductase	-1.06	1.77	53.78	Yes
PEG05649	AdoMet-dependent rRNA methyltransferase spb1	-1.06	1.15	30.34	Yes
PEG03382	Small nucleolar ribonucleoprotein complex subunit	-1.05	1.16	14.85	Yes
PEG07183	U3 small nucleolar ribonucleoprotein subunit	-1.04	1.14	17.00	Yes
PEG06633	Small nucleolar ribonucleoprotein complex subunit	-1.04	1.52	14.06	Yes
PEG09831	Aldehyde dehydrogenase	-1.04	3.72	0.40	No
PEG08909	Nuclear protein involved in pre-rRNA processing	-1.04	1.37	12.55	Yes
PEG06033	RRNA maturation protein	-1.04	1.41	10.79	No
PEG11244	DEAD/DEAH box helicase	-1.03	1.33	9.64	Yes
PEG02369	Nucleolus protein required for cell viability	-1.03	1.45	90.48	No
PEG00745	CCAAT-box-binding transcription factor	-1.02	1.44	16.17	Yes
PEG04694	Ribosome biogenesis	-1.02	1.61	21.29	Yes
PEG00786	GTP binding protein	-1.02	1.16	28.95	Yes
PEG04028	Antibiotic biosynthesis monooxygenase family protein	-1.01	1.45	148.07	Yes
PEG05814	Ribosome biogenesis protein	-1.01	1.04	28.93	Yes

[a]Accession number from the annotated genome sequence of *P.expansum* T01 (accession no. GCA_001008385.1).

[b]FPKM mean represents the average gene expression level in WT when exposed to H_2O_2.

[c]'Yes' indicating at least one putative PeAP1 binding site was identified in the promoter region of the gene while 'No' stand for none putative PeAP1 binding site was identified in the promoter region.

TABLE S3 Sequence of primer pairs used in the RT-qPCR analysis of downregulated genes in the *PeAP1* mutant.

**Gene ID**	**Annotation**	**Primer sequences (5' - 3')**
**ROS detoxification**		
PEG03317	Peroxiredoxin (*POD1*)	GGGAATGTTGCCAAGATGGA / TCAACCTCCTCCCTGGTCAA
PEG08064	Catalase-peroxidase (*CPX*)	GTCAACAACCCAGCCCAGTT / CCATTCTGGGCACTGTTGAA
PEG09232	Catalase (*CAT1*)	ACGCTCCACACCCTTCTTTG / TGCACACCGAATCCATCAAC
PEG06993	Catalase (*CAT2*)	TGCTAATGAGGCTGTGCCATT / TGAGCGGAGAACCACTTTGC
PEG08479	Glutathione peroxidase (*GSH-Px*)	GGCTCGGACGATGATATCCA / TTCACATCCAGTTTTCCGAGAA
PEG05350	Peroxiredoxin (*POD2*)	TACAACGATGCCTACGTCATGAG / GGGTCGGAGAGGAAGAGAATATC
PEG04985	Superoxide dismutase (*SOD*)	CCCCGAACATGACCCTCAT / TCCCGCTTGACGGATAGTTG
**Antioxidants biosynthesis**		
PEG02184	NADPH dehydrogenase (*NDH1*)	CCCCACTCGAGACATTGCTT / TTTCATTGGCAATCCGGAAT
PEG08383	Glutathione S-transferase (*GST1*)	ACAGCGACCGGGTACTGAGA / CAGCCGCCTTATCCTTGATG
PEG02421	Thioredoxin (*TRX1*)	CGAGCGATAACCACTCACCAA /GCCGGGTTGAGATAGAATGC
PEG08885	NADH oxidase (*NOX1*)	AATCCGAAGCCGCAGTTTG / CAGTGGTCGGCTCAACCAA
PEG05285	Glutathione S-transferase (*GST2*)	CGACAACTCCCCATTTACGAA / CGTAGGCGGCAATGATGTAAT
PEG10835	NADH oxidase (*NOX2*)	GGAGCTGATCCACGCCTACA / CGGCATCGATCATGACATTG
PEG08126	Glutathione S-transferase (*GST3*)	AACGCGTGACGGGTGTACTT / CCAGGGACCATCCCCAATAT
PEG08947	Glutaredoxin (*GRX*)	ACCGTGCCCAACATTTTCAT / AGCTCCTTCAGGTCCTTCTTACC
PEG05529	Thioredoxin (*TRX2*)	CCGACGTCCAGTTCTACAAGGT / AAGGTAGGCATGGCAGAGACA
PEG04883	Glutathione S-transferase (*GST4*)	CTCTCTCACCTTCCTCGATTCC / TGATATCGGCCATGTTGATCTT
PEG09485	Glutathione reductase (*GR*)	CTGCGCAAATTCGACCCTAT / TGGTTGCGGTGAAGGTTGA
PEG08441	Glutathione S-transferase (*GST5*)	TTTTCGAGAGCGGAGCAATC / GGTGCCAGGAGCGTAACTGA
PEG04204	Thioredoxin reductase (*TRXR*)	CCCGGTACCAGCTTCACTAACA / GTGATAGCCTGGCGGTAACG
**Sulfur metabolism**		
PEG06528	Sulfate permease (*PesB*)	CTTTGCCGAAGGCTTCAATT / GGCGGGTGCTCTTGAATATG
PEG10556	Sulfate adenylyltransferase (*PeSat*)	AGATTCTGCGCGAGTCCAA / CGGTCAGGAAGATGGTGAAAC

TABLE S4 Sequence of primer pairs used for gene knockout and complementary vector construction of genes regulated by PeAP1.

Gene		Primer sequences (5' - 3')
**Gene knockout vector construction**		
PePrx1	up	CTAAGGTTGTTACTACCAGAGAAAA / GGTTGCAGTAGGTATAATATTGATG
	down	GGCACAGTGTCTTAAAGCCAG / GAGACAAAGTTACTAATAGCCCG
PePrx2	up	AGTTCCTTTGATCATATTCATTGT / TTTGAAGATTGAAAGTATTGAAGAG
	down	CAAGCAGCTGTAGATTCACTCGT / CTTGTACAGGCAAAACACTCTCAT
PeGSH-Px	up	GCTACCTTTGTGTTGTATGACTTC / GATTGTTATTTGACGTGAATAGGT
	down	GGCTGTGCTCGAGGTGTTCTTA / GCAGACCAACATCCCTTCTACAA
PeSOD	up	ATCACTAGGAATACACTATCGGAAC / CGTCTGGATATAAGAATCGCAT
	down	ATATGTAACCAGATCAATCGCC / CCAATACCAGTCAACACAGTCC
PeCPX	up	ACCTCAGACCCATACTCTTACAAT / GATGAAAGTTGTTATGGTGAATCA
	down	ATAATTTGTCGATTACTATATGATA / GATATATTTGACATTGTCTTTTG
PeCAT1	up	CTAAAAATGGTAAGAGATGTGCG / TGCGACAGTTAAATGATATAACTGT
	down	TAGACGATGTCCGTTATGACTGTAT / CAGAAGTCGATACCACCATTTATTA
PeGSH5	up	CTGGGTTTATCGTCCATTCTG / GGTAGAATATGGGTAGAATCTTGG
	down	TTGACGGGAGTATCTTAGGTTTC / CCTACTGGAAGTTTGCGGTCT
PeGSH1	up	AAGAAAGTCAATCGACAGTATAAAG / ATTGATAGAACAAGAAATTGGACT
	down	AGAAGAATTGCTAAGGCATTGATA / GGAATGTTATTGGAGCCAGTTA
PeGSH3	up	GTAGAAGGAGACATAGGACCCGAA / TGTTGCGATTTAAGTGTGATTGAA
	down	TCGGTTGGGACTGCTATGGA / CTGGCTACGCCCCTACATCTC
PeGRX	up	CACTCCACAACCATCTGTCCAA / GAGCCGAAAAGACGGGTGAA
	down	ATCTAAGCGGTGATTTCACACA / TCCTTCCTCTCAGTCCAAGTCTA
PeTRX1	up	ACCACCAATACTCTAATACAGGCTC / TGTGTTAAGGACGTAGGAGGAGA
	down	AGAGTATCGTCATGGGAATTTC / ACTTCTGTGTCTGGTCCTCAAT
PeTRX2	up	ATTGAACTAAGGTGTTGGCTGTA / TTTGAGAGTAGAAGGCGTGAAG
	down	ATTTGAAAATTGAGATTCGCGC / TACTTGATGTCGCAGTGGGCT
**PCR detection**		
PePrx1		CTAAGGTTGTTACTACCAGAGAAAA / GAGACAAAGTTACTAATAGCCCG
PePrx2		AGTTCCTTTGATCATATTCATTGT / CTTGTACAGGCAAAACACTCTCAT
PeGSH-Px		GCTACCTTTGTGTTGTATGACTTC / GCAGACCAACATCCCTTCTACAA
PeSOD		ATCACTAGGAATACACTATCGGAAC / CCAATACCAGTCAACACAGTCC
PeCPX		ACCTCAGACCCATACTCTTACAAT / TTCCTTTGCCCTCGGACGAG
PeCAT1		CTAAAAATGGTAAGAGATGTGCG / TTCCTTTGCCCTCGGACGAG
PeGSH5		CTGGGTTTATCGTCCATTCTG / CCTACTGGAAGTTTGCGGTCT
PeGSH1		AAGAAAGTCAATCGACAGTATAAAG / GGAATGTTATTGGAGCCAGTTA
PeGSH3		GTAGAAGGAGACATAGGACCCGAA / CTGGCTACGCCCCTACATCTC
PeGRX		CACTCCACAACCATCTGTCCAA / TCCTTCCTCTCAGTCCAAGTCTA
PeTRX1		ACCACCAATACTCTAATACAGGCTC / ACTTCTGTGTCTGGTCCTCAAT
PeTRX2		ATTGAACTAAGGTGTTGGCTGTA / TACTTGATGTCGCAGTGGGCT

Complementary vector construction

PePrx1	CTAAGGTTGTTACTACCAGAGAAAA / GAGACAAAGTTACTAATAGCCCG
PePrx2	AGTTCCTTTGATCATATTCATTGT / CTTGTACAGGCAAAACACTCTCAT
PeGSH-Px	GCTACCTTTGTGTTGTATGACTTC / GCAGACCAACATCCCTTCTACAA
PeSOD	ATCACTAGGAATACACTATCGGAAC / CCAATACCAGTCAACACAGTCC
PeCPX	ACCTCAGACCCATACTCTTACAAT / GATATATTTGACATTGTCTTTTG
PeCAT1	CTAAAAATGGTAAGAGATGTGCG / CAGAAGTCGATACCACCATTTATTA
PeGSH5	CTGGGTTTATCGTCCATTCTG / CCTACTGGAAGTTTGCGGTCT
PeGSH1	AAGAAAGTCAATCGACAGTATAAAG / GGAATGTTATTGGAGCCAGTTA
PeGSH3	GTAGAAGGAGACATAGGACCCGAA / CTGGCTACGCCCCTACATCTC
PeGRX	CACTCCACAACCATCTGTCCAA / TCCTTCCTCTCAGTCCAAGTCTA
PeTRX1	ACCACCAATACTCTAATACAGGCTC / ACTTCTGTGTCTGGTCCTCAAT
PeTRX2	ATTGAACTAAGGTGTTGGCTGTA / TACTTGATGTCGCAGTGGGCT
